# Genomic studies in *Linum* shed light on the evolution of the distyly supergene and the molecular basis of convergent floral evolution

**DOI:** 10.1111/nph.70392

**Published:** 2025-07-18

**Authors:** Panagiotis‐Ioannis Zervakis, Zoé Postel, Aleksandra Losvik, Marco Fracassetti, Lucile Solér, Estelle Proux‐Wéra, Ignas Bunikis, Allison Churcher, Tanja Slotte

**Affiliations:** ^1^ Department of Ecology, Environment and Plant Sciences Science for Life Laboratory, Stockholm University 106 91 Stockholm Sweden; ^2^ Department of Medical Biochemistry and Microbiology Uppsala University, National Bioinformatics Infrastructure Sweden (NBIS), Science for Life Laboratory, Uppsala University 751 23 Uppsala Sweden; ^3^ Department of Biochemistry and Biophysics National Bioinformatics Infrastructure Sweden, Science for Life Laboratory, Stockholm University 171 65 Solna Sweden; ^4^ Department of Immunology, Genetics and Pathology Uppsala Genome Center, Science for Life Laboratory, Uppsala University 751 23 Uppsala Sweden; ^5^ Department of Plant Physiology National Bioinformatics Infrastructure Sweden (NBIS), SciLifeLab, Umeå University 901 87 Umeå Sweden

**Keywords:** balancing selection, brassinosteroid pathway, floral evolution, functional constraint, hemizygosity, heterostyly, mating system, structural variation

## Abstract

- Distyly, an example of convergent evolution, is governed by a supergene, the *S‐*locus, in several species. Recent studies highlight similar genomic architectures of independently evolved *S*‐loci, but its mode of origin and whether similar regulatory pathways underlie the convergent evolution of distyly remains unclear.
- We examined the evolution of supergenes and mechanisms underlying distyly in *Linum* species that diverged *c*. 33 million years ago (Ma). Using haplotype‐resolved genomes and population genomics, we identified and characterized the *S‐*loci of *Linum perenne* (distylous) and *Linum grandiflorum* (style length dimorphic), and compared them to that of *Linum tenue* (distylous). We then tested for a conserved hormonal mechanism regulating style length polymorphism in *Linum*.
- The *S*‐locus supergene was consistently hemizygous in short‐styled individuals across all three species, although it showed variation in size, gene content, repeat elements and extent of recombination suppression. Two *S‐*linked candidate genes, *TSS1* (style length) and *WDR‐44* (anther height/pollen self‐incompatibility), were conserved. Consistent with a brassinosteroid‐dependent role of *TSS1*, epibrassinolide treatment revealed a conserved, morph‐specific effect on style length.
- *S‐*locus structural polymorphism, candidate distyly genes and mechanisms regulating style length remain conserved > 30 Ma in *Linum*. In combination with findings from other systems, our results suggest that the brassinosteroid pathway frequently contributes to style length polymorphism.

## Introduction

Distyly is a floral polymorphism that promotes outcrossing and is recognized as a prominent example of convergent floral evolution (Barrett, 2019). In distylous species, there are two floral morphs that differ reciprocally in the positions of anthers and stigmas. Long‐styled (L‐morph or pin) plants have stigmas in a high position in the flower, and anthers in a low position, whereas short‐styled (S‐morph or thrum) individuals have the opposite arrangement. Differences in flower structure are usually accompanied by differences in pollen and stigma traits and by heteromorphic self‐incompatibility (SI), which limits self‐ and intra‐morph pollination. Distyly has evolved independently multiple times in flowering plants (Lloyd & Webb, 1992; Naiki, 2012), suggesting that it provides a solution to a common set of selective pressures (Shore *et al*., 2019; Simón‐Porcar *et al*., 2024). Specifically, reciprocal morph differences increase the precision of pollen transfer by pollinators (Darwin, 1877; Lloyd & Webb, 1992; Barrett, 2019; Simón‐Porcar *et al*., 2024), whereas heteromorphic SI confers inbreeding avoidance (Charlesworth & Charlesworth, 1979).

In distylous species where genetic studies have been done, distyly is governed by a single Mendelian locus, the *S‐*locus, with one dominant and one recessive allele (Bateson & Gregory, 1905; Laibach, 1923; reviewed by Ganders, 1979), which controls both floral morphology and heteromorphic SI. In most systems, the L‐morph is homozygous for the recessive *s‐*allele (*s/s*), whereas the S‐morph is genetically heterozygous (*S/s*; reviewed by Ganders, 1979). To explain how a single Mendelian locus could control this multi‐trait balanced polymorphism, Ernst (1936) proposed that in distylous *Primula*, the *S‐*locus harbored at least three separate and polymorphic genes, present in close linkage and controlling different aspects of distyly. Under Ernst's model, the *S*‐locus constitutes a supergene, defined as ‘a system of closely linked loci controlling a polymorphic phenotype, such that a non‐recombining genome region is structured into two or more distinct haplotypes, each carrying a set of alleles that control multiple aspects of one of the phenotypes’ (Charlesworth, 2016).

Genomic characterization of independently evolved distyly *S‐*loci support this model, revealing multiple closely linked genes important for trait polymorphism, consistent with a supergene architecture (e.g. *Fagopyrum* (Yasui *et al*., 2012; Fawcett *et al*., 2023); *Primula* (Huu *et al*., 2016; Li *et al*., 2016); *Turnera* (Shore *et al*., 2019); *Linum* (Gutiérrez‐Valencia *et al*., 2022); *Nymphoides* (Yang *et al*., 2023); *Gelsemium* (Zhao *et al*., 2023); Oleaceae (Castric *et al*., 2024; Raimondeau *et al*., 2024); *Mussaenda* (Yuan *et al*., 2025)). However, unlike other types of supergenes, which often harbor inversions, all distyly supergenes studied in detail so far instead harbor large indels (reviewed in Gutiérrez‐Valencia *et al*., 2021b). The supergene is usually hemizygous in S‐morph individuals, with S‐morph‐specific expression of dominant *S‐*linked genes that control floral morph and are absent from the allelic genome location in the L‐morph (reviewed in Gutiérrez‐Valencia *et al*., 2021b). Hemizygosity ensures both dominant expression and absence of recombination between the recessive and dominant alleles at the distyly supergene.

While an increasing number of distyly supergenes have been characterized, many fundamental questions about their evolution remain unanswered. For instance, while gene sets at distyly supergenes have been assembled stepwise via gene duplication (e.g. *Primula* (Huu *et al*., 2020), *L. tenue* (Gutiérrez‐Valencia *et al*., 2022)), it remains unclear whether this process continues after supergene formation and what the mode of origin of distyly supergenes is, limiting our understanding of the role of gene duplication and potential gene loss in this process. Another open question concerns the extent to which repeated evolution of similar genomic architectures of the distyly supergenes across different families is accompanied by functional similarities in the mechanisms regulating distyly, especially given that independently evolved *S‐*loci do not share orthologous genes. Functional analyses have demonstrated that *S‐*linked genes involved in brassinosteroid inactivation control style length and female SI in at least two distylous systems (in *Primula* (Huu *et al*., 2016, 2022) and in *Turnera* (Shore *et al*., 2019; Matzke *et al*., 2020, 2021)). Studies in additional distylous systems are required to determine the mode of origin of the *S*‐locus and whether distyly is generally accompanied by parallel evolution at the biochemical pathway level, and ultimately improve our understanding of distyly supergene evolution.

**Fig. 1:**
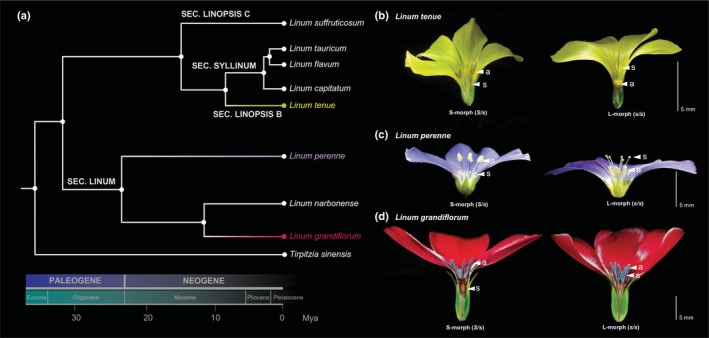
Schematic phylogeny and floral morphs of the study species. (a) Schematic phylogeny and divergence times of the species used in the study. The three main species of interest (*Linum grandiflorum*, *L. perenne* and *L. tenue*) are highlighted with color. Timeline at the bottom was based on Maguilla *et al*. (2021). SEC.: Section (b) Floral morph of S‐morph (left) and L‐morph (right) *L. tenue*. (c) Floral morph of S‐morph (left) and L‐morph (right) *L. perenne*. (d) Floral morph of S‐morph (left) and L‐morph (right) *L. grandiflorum*. In panels b–d, positions of anthers (a) and stigmas (s) are marked in the figure and magnification is indicated by a scale bar, 5 mm. Part of the corolla and sepals were removed for improved visibility of sexual organ location.

*Linum* (wild flaxseed species) is a classic system for the study of the function, evolution and genetic basis of distyly (Darwin, 1863, 1877; Dulberger, 1992; Armbruster *et al*., 2006; Fig. 1). This system is of particular interest due to its polymorphisms in style length, including distyly (Fig. 1b,c), and stigma height dimorphism (Fig. 1d), with variation in ancillary floral traits (Armbruster *et al*., 2006; McDill *et al*., 2009; Ruiz‐Martin *et al*., 2018; Maguilla *et al*., 2021). The presence of varied stylar polymorphisms, as well as recurrent loss of distyly, makes *Linum* a particularly suitable system for dissecting the genetic basis of distyly. Building on a high‐quality genome assembly, we recently characterized the distyly *S‐*locus of the distylous and self‐incompatible *Linum tenue* Desf. (Fig. 1b) and showed that it constitutes a supergene which harbors a *c*. 260‐kb indel as well as a *c*. 15‐kb *S*‐linked region, rendering the S‐morph predominantly hemizygous (Gutiérrez‐Valencia *et al*., 2022). The *L. tenue S‐*locus harbors nine protein‐coding genes (seven of which were found only in the *S*‐allele while the remaining two were shared between the two haplotypes), including hemizygous candidate genes for style length (*THRUM STYLE SPECIFIC 1* or *LtTSS1*, hereafter called *TSS1*) and anther height/pollen SI type (*LtWDR‐44*, hereafter called *WDR‐44*). In the closely related selfing species *L. trigynum*, which recently lost distyly and is homostylous, that is, monomorphic with anthers and stigmas at the same height, *WDR‐44* is present but expressed at a lower level than in SI *L. tenue* thrums (Gutiérrez‐Valencia *et al*., 2024). Altered expression of *WDR‐44* is associated with a switch in pollen SI function from thrum‐to‐pin‐type enabling self‐compatibility (SC), and with a reduction in anther height, suggesting a role for this gene in pollen SI and floral morphology (Gutiérrez‐Valencia *et al*., 2024). However, it is not currently clear whether *TSS1* and *WDR‐44* are generally important for distyly in *Linum*, as ancestral state reconstruction suggested that divergent *Linum* species may have independently evolved distyly (Armbruster *et al*., 2006; McDill *et al*., 2009; Ruiz‐Martin *et al*., 2018).

To help further understand the origin, evolution and composition of the distyly *S‐*locus, we used newly built high‐quality haplotype‐resolved genome assemblies of two *Linum* species: the distylous *L. perenne* and the style length dimorphic *L. grandiflorum*, which diverged from each other *c*. 18 million years ago (Ma) and from *L. tenue c*. 33 Ma (Maguilla *et al*., 2021; Fig. 1a). Like *L. tenue*, both *L. perenne* and *L. grandiflorum* have heteromorphic SI (Murray, 1986), but *L. grandiflorum* lacks anther height dimorphism. We first identified and characterized structural variation, gene and repeat content, and linkage disequilibrium (LD) at the *S‐*loci of *L. grandiflorum* and *L. perenne* in comparison with that of *L. tenue*. Based on these results, we then tested whether ongoing gene duplication has contributed to the assembly of the *S*‐locus and inferred the origin of the distyly *S‐*locus in *Linum*. Finally, we experimentally tested whether downregulation of brassinosteroid‐responsive genes by the distyly supergene is a conserved mechanism that controls style length polymorphism in *Linum*. Our results help bridge gaps in understanding the origin and evolution of distyly supergenes, and whether this iconic case of floral convergent evolution is accompanied by parallel evolution at the biochemical pathway level.

## Materials and Methods

### Biological material for genome assembly and annotation

For *de novo* genome assembly of *Linum perenne* L. and *L. grandiflorum* Desf., we snap‐froze leaves from one S‐morph individual of *L. perenne* IPK Gatersleben accession LIN 2003 (here named L96A) and one S‐morph individual of *L. grandiflorum* accession LIN 10 (here named L62.06; Supporting Information Table S1). For annotation of genome assemblies, we snap‐froze at least two replicates each of leaves, stems, early and late flower buds (collected at two stages for *L. perenne* and at three stages for *L. grandiflorum*) and open flowers of *L. perenne* L96A and *L. grandiflorum* L62.06 for RNA extraction and sequencing.

### Plant growth conditions

Seeds were surface‐sterilized, sown on sterile plates with half‐strength Murashige & Skoog medium (Sigma‐Aldrich) and stratified and moved to standard long‐day conditions (16 h 120 μE light at 20°C : 8 h dark at 18°C, 60% maximum humidity) until seedlings emerged. Seedlings were transplanted to pots containing a mixture of soil (Hasselfors Garden, Sweden) and gravel (1.5 : 1), with the addition of perlite and vermiculite. *Linum perenne* plants were vernalized for 9 wk under short‐day conditions (8 h 110 μE light at 6°C, 16 h dark at 2°C, 65% maximum humidity), with transition conditions in place 2 wk before and after vernalization (11 h 120 μE light at 15°C, 13 h dark at 10°C, 65% maximum humidity).

### High molecular weight DNA isolation

High molecular weight (HMW) DNA was extracted using a two‐step protocol from a total of 2 g fresh‐frozen leaves. We followed a modified protocol from (Fulton *et al*., 1995) with purification using Genomic‐Tip/500 (Qiagen, Hilden, Germany). HMW DNA quality was checked spectrophotometrically and through pulsed‐field gel electrophoresis using SeaKem Gold agarose (Lonza, Rockland, ME, USA), 0.5X KBB buffer (Sage Science) and a Pippin Pulse Electrophoresis Power Supply System (Sage Science, Beverly, MA, USA), with poststaining using GelRed (Biotium, Fremont, CA, USA).

### PacBio high‐fidelity sequencing

HMW DNA was used to generate SMRTBell libraries for high‐fidelity (HiFi) long‐read sequencing. Each library was sequenced on two SMRT cells in HiFi mode on a Sequel II (Pacific Biosciences), which resulted in 31 and 50 Gbases of HiFi data for *L. perenne* and *L. grandiflorum*, respectively, with an insert size of 15 kbp.

### Hi‐C data generation

To generate high‐quality proximity ligation libraries (Hi‐C) for scaffolding of genome assemblies, a total of 300 mg of fresh‐frozen leaf tissue was first ground to a fine powder in liquid nitrogen. Hi‐C libraries were generated using the Dovetail OmniC Kit. Sequencing on an Illumina NovaSeq6000 generated a total of 1.0×10^9^ paired‐end 150‐bp reads for *L. perenne*, and 2.3×10^9^ paired‐end 150‐bp reads for *L. grandiflorum*.

### RNA extraction and sequencing

For genome annotation, we obtained RNA sequencing data from leaves, stems, flower buds and open flowers of *L. perenne* L96A and *L. grandiflorum* L62.06. Total RNA was extracted using the RNeasy Plant Mini Kit (Qiagen). Sequencing libraries were prepared using the TruSeq stranded mRNA Library Preparation Kit (Illumina, San Diego, CA, USA), including polyA selection and unique dual indexes (Illumina), and were sequenced using paired‐end 150‐bp reads on a NovaSeq6000 system.

### *De novo* genome assembly

We generated primary and haplotype‐resolved genome assemblies based on HiFi and Hi‐C data of our outbred S‐morph *L. perenne* L96A and *L*. *grandiflorum* L62.06 individuals using integrated Hi‐C assembly settings in Hifiasm (Cheng *et al*., 2021). For each species, we generated two high‐quality phased haplotype assemblies, designated as hap1 and hap2, as well as a primary assembly. Assembly completeness was checked using Benchmarking Universal Single‐Copy Orthologs (BUSCO; Waterhouse *et al*., 2018) against the eudicots_odb10 gene dataset. Before annotation, assemblies were screened for contamination (Notes S1) and presence of chloroplast or mitochondrial sequences as described in Gutiérrez‐Valencia *et al*. (2024).

### Genome annotation

Annotation of genes and repeats was performed using open‐source pipelines in use at the National Bioinformatics Infrastructure Sweden (NBIS) Annotation and Assembly unit (See the Data Availability section). We used a combination of evidence‐based and *ab initio* annotation, followed by functional annotation. In addition, repeats were modeled and annotated after vetting them against annotated genes. We fully annotated the primary and haplotype‐resolved assemblies of each species.

For evidence‐based gene annotation methods, we used both proteins and transcriptomes. As protein evidence, we used proteins from sequenced *Linum* species (*Linum tenue* Desf., *Linum usitatissimum* L.), more distantly related species from the Malpighiales (*Manihot esculenta* Crantz, *Populus trichocarpa* Torr. & A. Gray ex Hook, *Ricinus communis* L. *and Salix purpurea* L.), the Vitales (*Vitis vinifera* L.) and Uniprot data for rosids. We further used transcriptome data from leaves, stems, buds and flowers of *L. perenne* L96A and *L. grandiflorum* L62.06. After adapter‐trimming with fastp v.0.23.2 (Chen *et al*., 2018), RNAseq reads were aligned to the reference genome using Hisat2 v.2.1.0 (Kim *et al*., 2015). Genome‐guided assembly of transcripts was done using StringTie v.2.2.1 (Pertea *et al*., 2015), using MultiQC (Ewels *et al*., 2016) for quality‐checking. Evidence‐based annotation was performed using maker v.3.01.02 (Holt & Yandell, 2011), including aligned transcript sequences and reference proteins as evidence, whereas *ab initio* training was conducted using GeneMark v.4.3 (Besemer *et al*., 2001), Augustus v.3.3.3 (Stanke *et al*., 2006) and Snap 2013_11_29 (Korf, 2004). Finally, results from *ab initio* and evidence‐based annotation were combined to produce final gene builds, which were functionally annotated using Blast (v.2.9.0; Altschul *et al*., 1990) matches against Uniprot/Swissprot and results from InterproScan v.5.59‐91.0 (Hunter *et al*., 2012).

Species‐specific repeat libraries were generated using RepeatModeler (Smit & Hubley, 2008), and candidate repeats were vetted against protein evidence (excluding transposons) to exclude low‐complexity coding sequences. Finally, repeat identification was performed using RepeatMasker (Smit *et al*., 2013) and RepeatRunner (Smith *et al*., 2007).

### Manual curation of annotation in the *S*‐locus region

To describe and compare the gene content of the *S‐*locus region across species, we manually curated gene annotation in genome regions of *L. perenne* and *L. grandiflorum* containing their respective S‐morph hemizygous *S*‐locus (*L. perenne* h1tg000002l: 1080000‐4890 000; *L. grandiflorum* h1tg000023l: 11240000‐12420000 – see section ‘Identification of the *S‐*locus in *L. perenne* and *L. grandiflorum*’ below for details) by inspecting transcriptome evidence for the original annotation as well as for TransDecoder/StringTie v.2.2.1‐based gene predictions. Manual curation resulted in removal of eight gene models and addition of two gene models in the *L. perenne* hemizygous *S*‐locus region, and removal of 14 gene models that were not supported by transcriptome data, and addition of five new gene models based on TransDecoder (https://github.com/TransDecoder/TransDecoder) output in the *L. grandiflorum* hemizygous *S‐*locus region.

We performed additional repeat annotation to improve transposable element (TE) classification completeness before tests for repeat enrichment at the *S‐*locus. Specifically, we used HiTE v.3.2 (Hu *et al*., 2024) in conjunction with LTR_retriever v.2.9.9 (Ou & Jiang, 2018) to build a repeat element library and annotate the genome with full‐length TEs, classified using RepeatMasker v.4.1.5 (Smit *et al*., 2013) in sensitive mode. Statistical comparison between the *S‐*locus and genome‐wide repeat content was done using binomial tests in R v.4.3.2.

### Whole‐genome short‐read sequencing

DNA for short‐read sequencing was extracted from 157 samples of *L. perenne* from three natural populations and two accessions, and for *L. grandiflorum*, we extracted DNA from 22 individuals from three accessions (Table S1), using the *Quick*‐DNA Miniprep Plus Kit (Zymo Research, Irvine, CA, USA). We also acquired short‐read sequencing data for five additional distylous species of *Linum* (Fig. 1; Table S1) following the same procedure but using magnetic beads and the Quick‐DNA MagBead Plus Kit (Zymo Research) for DNA extraction. Sequencing libraries were prepared from 1 μg DNA using the TruSeq PCR‐free DNA sample preparation kit (Illumina) with unique dual indexes, targeting an insert size of 350 bp. Libraries were sequenced on a NovaSeq 6000 system, yielding paired‐end 150 bp reads.

### Short‐read processing, mapping, variant calling and filtering

Illumina whole‐genome resequencing reads were adapter‐ and quality‐trimmed using BBDuk from BBMap v.38.61b (Bushnell, 2014), and mapped using BWA‐MEM v.0.7.18 (Li, 2013). We excluded mapped reads with a mapping quality lower than 20 and duplicates using Picard tools v.3.1.1 (http://broadinstitute.github.io/picard). Variants were called using BCFtools *mpileup* v.1.17 (Danecek & McCarthy, 2017) independently for each species. We kept only bi‐allelic variants and invariant sites, and applied additional filters for depth, missingness and mapping quality (BCFtools *min_depth* = 5; *max_depth* = 200; *missingness* = 0.9; *min_quality* = 20). Due to the high repeat content of our assemblies, additional masking of repeats was necessary. Hence, we masked repeats using ‘*bedtools intersect*’ and by filtering on coverage as in Gutiérrez‐Valencia *et al*. (2021a). Finally, to reduce false heterozygous calls, we applied an allele balance filter with thresholds 0.2 and 0.8, setting heterozygous calls that failed this criterion to missing.

### Identification of the *S*‐locus in *L. perenne* and *L. grandiflorum*

To identify the *S‐*locus we tested for an association between single‐nucleotide polymorphism (SNP) genotype and floral morph using genome‐wide association mapping (GWAS). Before GWAS, we removed sites with missing data, rare variants (minor allele frequency < 0.05) and pruned variants with high LD (*r* ^*2*^ > 0.2) in 50‐kb windows. We performed association analysis in plink v.1.90b4.9 (Purcell *et al*., 2007) using Fisher's exact test on genotypes, assuming a dominant effect for the minor allele and applying a false discovery rate (FDR) *P*‐value adjustment. In *L. grandiflorum*, this association analysis used 15 927 LD‐pruned SNPs in 11 S‐morph and 7 L‐morph individuals from three accessions (Table S1). In *L. perenne*, we first analyzed 13 992 LD‐pruned SNPs genome‐wide from 7 S‐morph and 12 L‐morph *L. perenne* individuals from one family. Because family‐based analyses can have limited resolution, we validated our findings by GWAS analyses on 53 individuals from one natural population (ger3, Table S1; Notes S2).

We performed depth of coverage analyses to identify genomic regions with presence–absence variation between morphs in the genomes of *L. perenne* and *L. grandiflorum* and narrow down the position of the *S‐*locus. Depth of coverage of reads mapped to the hap1 haplotype‐resolved assembly of each species was calculated in 300‐kb windows using BEDTools v.2.31.1 (Quinlan & Hall, 2010) and normalized by total sample read count. We identified windows that differed in normalized median coverage between individuals with different floral morphs (L‐ vs S‐morph) using a two‐sample Fisher–Pitman permutation test in R (v.4.3.2, package ‘coin’ v.1.4.3), with 1 000 000 permutations, using a significance threshold of *P* ≤ 0.01 after Bonferroni multiple testing correction.

Finally, we tested for limited recombination in a large region around the S‐hemizygous region of *L. perenne*, where widespread GWAS hits were present. These analyses were conducted as described in Notes S3, on population genomic data from three natural populations of *L. perenne* from Germany (Table S1).

### Stepwise assembly of the S‐locus gene set

We estimated *d*_*S*_ between *S‐*locus genes and their closest paralogs identified by OrthoFinder v.2.5.5 (Emms & Kelly, 2019) in *L. grandiflorum* and *L. perenne*, to test for an impact of stepwise gene movement on *S‐*locus gene content. We used the Nei–Gojobori model in MEGA X (Tamura *et al*., 2021) to estimate *d*_*S*_. Widely different *d*_*S*_ estimates for different genes imply stepwise gene duplication at different times, and very low estimates suggest very recent gene duplication.

### Selection on *S*‐locus candidate genes and the age of the *S*‐locus in *Linum*

To assess purifying selection on *S‐*locus candidate genes *TSS1* and *WDR‐44*, we estimated the ratio of nonsynonymous to synonymous sequence divergence (*d*_*N*_ */d*_*S*_) for both genes across *L. perenne*, *L. grandiflorum*, *L. tenue*, and five additional distylous species of *Linum* (Fig. 1; Table S1). We obtained *TSS1* and *WDR‐44* sequences of the additional distylous *Linum* using the HybPiper pipeline (Johnson *et al*., 2016), based on a target file, including available gene sequences of *TSS1* and *WDR‐44* from *L. tenue* and *L. trigynum* (Gutiérrez‐Valencia *et al*., 2022, 2024), *L. grandiflorum* and *L. perenne*. We constructed multiple sequence alignments for coding sequences of each gene using T‐Coffee (Notredame *et al*., 2000), inferred gene trees using RAxML with the GTR‐Gamma model of nucleotide substitution (Stamatakis, 2014) and estimated *d*_*N*_ */d*_*S*_ ratios using codeml in PAML (Yang, 2007), comparing a model allowing different values of *d*_*N*_ */d*_*S*_ for each branch to a constrained model with only one *d*_*N*_ */d*_*S*_ ratio for the whole tree using a likelihood ratio test (LRT).

Next, we took advantage of the presence of *WDR‐44* paralogs to compare selective pressures on the *S*‐locus sequence compared with its paralogs and to infer the timing of duplication. To compare selective pressures on the *S‐*locus vs paralogs of WDR‐44, we used the codeml branch model and three phylogenetic tree annotations: (1) only one *d*_*N*_ */d*_*S*_ ratio for the whole tree, (2) one *d*_*N*_ */d*_*S*_ ratio for *S*‐locus sequences and one for paralogs, and (3) one *d*_*N*_ */d*_*S*_ ratio per species, with model selection based on LRTs. We tested for positive selection at the branch separating the *S*‐locus copy of *WDR‐44* from its paralogs by conducting an additional LRT using the same dataset. To estimate the timing of duplication of *WDR‐44*, we ran beast2 v.2.7.7 (Bouckaert *et al*., 2019) with a lognormal optimized relaxed molecular clock and a calibrated Yule model. For calibration, we used the timing of diversification of *Linum* species, that is *c*. 33 Ma (parameters: lognormal, M = 3.5, S = 0.05; Maguilla *et al*., 2021). We set the chain length to 100 000 000, sampling every 10 000^th^ step and obtained the final estimate after excluding the first 10% of trees as burn‐in. We also applied a simple molecular clock analysis to estimate the divergence time of *L. tenue* and *L. perenne* (*t* = *d*_*S*_/(2*μ*)), based on synonymous sequence divergence at *TSS1* and *WDR‐44* estimated under the Nei–Gojobori model in MEGA X and assuming a mutation rate (*μ*) of 7×10^−9^ (Ossowski *et al*., 2010). Finally, we included *WDR‐44* sequences for three outgroups in our phylogenetic reconstruction. For *Tirpitzia sinensis* (Linaceae), the *WDR‐44* sequence was retrieved using the same procedure as for the *Linum* species. For *Manihot esculenta* (Euphorbiaceae), we used *Manes.15G085300*, and for *Populus trichocarpa* (Salicaceae), *Potri.011G122500* as in Gutiérrez‐Valencia *et al*. (2022).

### Brassinosteroid supplementation experiment

To test whether supplementation with active brassinosteroid hormone affects style length specifically in the S‐morph of both *L. perenne* and *L. tenue*, we performed a controlled experiment with two treatments: epibrassinolide (eBL) treatment (10 μM 24‐eBL dissolved in 0.1% dimethylsulfoxide, DMSO) and control treatment (0.1% DMSO). eBL concentration was chosen based on initial tests with 1, 10 and 20 μM eBL. Young flower buds were injected until saturation on two consecutive days with either eBL or control solution. Fully open flowers were dissected, photographed under a stereo microscope (Leica S APO) and style and stamen lengths were determined using ImageJ v.1.53 k (Schneider *et al*., 2012). One to two flowers of eight L‐morph and 11 *L. perenne* S‐morph individuals were treated with either eBL or control treatment, for a total of 90 measurements of style and stamen length. For *L. tenue*, two flowers of each of 19 L‐morph and S‐morph individuals were subjected to each treatment type, for a total of 152 measurements of style and stamen length. The experiment was not performed on *L. grandiflorum* due to growth chamber space and time constraints.

Style and stamen lengths were analyzed separately using analysis of variance (ANOVA) in R v.4.1.1 using the lm() function, with organ length as the response and floral morph, hormonal treatment, and the interaction between floral morph and treatment as predictors. For models with significant effects, we conducted a *post hoc* test using the Tukey ‘Honest Significant Difference’ (HSD) method and obtained 95% confidence intervals (CIs) for the difference in mean organ length.

To test whether eBL treatment affects style cell length, we quantified cell length in styles after eBL or control treatment in *L. perenne*. To obtain an image of the epidermal cells in control and treated styles, a thin layer of UV‐cured transparent nail polish (Semilac, Poznan, Poland) was applied to a microscope slide and excised styles were carefully placed on the surface of the nail polish (this method was not feasible for fixed *L. tenue* material, and therefore, it was only performed in *L. perenne*). After hardening under the UV light, the imprint was photographed under a light microscope (Olympus BX60) and cell sizes were measured using ImageJ v.1.53k (Schneider *et al*., 2012). Cell length measurements were obtained separately for three different sections at the bottom, middle and top of the style (10 cells measured per section), following Ushijima *et al*. (2015) and Foroozani *et al*. (2023). Measurements were performed on two to four flowers of each of eight *L. perenne* L‐morph individuals and 11 S‐morph individuals, resulting in a total of 805 cell length measurements.

The 10 cell length measurements for each style section were averaged before linear model analysis in R v.4.1.1. We tested for an effect of eBL treatment on mean style cell length using a linear model with mean cell length as the response, and floral morph, style section (bottom, middle or top) and hormonal treatment as predictor variables. Cell lengths were log‐transformed to improve the normality of residuals. *Post hoc* tests were performed, and 95% CI intervals obtained for significant effects, as described above.

## Results

### High‐quality phased genome assemblies of *L. perenne* and *L. grandiflorum*

As both *L. grandiflorum* and *L. perenne* are SI and outbred, we assembled both a primary assembly and a pair of haplotype‐resolved assemblies for each species based on PacBio HiFi and Hi‐C data. We sequenced S‐morph individuals, which are expected to harbor both the dominant and recessive alleles at their *S‐*loci. We obtained highly complete primary and haplotype‐resolved assemblies with BUSCO scores ranging from 94.0% to 95.1% (Table S2) that were highly contiguous, with N50 scores ranging from 10.2 Mb to 69.9 Mb (Table S2). Assembly lengths were similar to genome sizes estimated by flow cytometry (Table S2).

We annotated the primary and haplotype‐resolved assemblies using a combination of *ab initio* and evidence‐based methods. We identified a total of *c*. 42 000 protein‐coding genes in our *L. grandiflorum* assemblies, whereas our *L. perenne* assemblies had *c*. 45 000 protein‐coding genes (Dataset S1A). Compared with *L. tenue*, where 49.4% of the genome consisted of repeats (Gutiérrez‐Valencia *et al*., 2022), the genomes of *L. grandiflorum* and *L. perenne* were richer in repeats, with 78.2% and 69.5% of the respective genome assemblies annotated as repetitive (Dataset S1A). The relatively high gene numbers of *L. perenne* and *L. grandiflorum* likely result from an ancient whole‐genome duplication in the ancestor of these species (Sveinsson *et al*., 2014).

### Hemizygosity in the *S*‐morph is a common feature of *Linum S*‐locus supergenes

To identify *S*‐loci in *L. perenne* and *L. grandiflorum*, we searched for SNPs whose genotypes were associated with floral morph. Because many distyly *S*‐locus supergenes harbor presence–absence variation, we also tested for presence–absence variation between floral morphs using short‐read depth of coverage analyses.

**Fig. 2:**
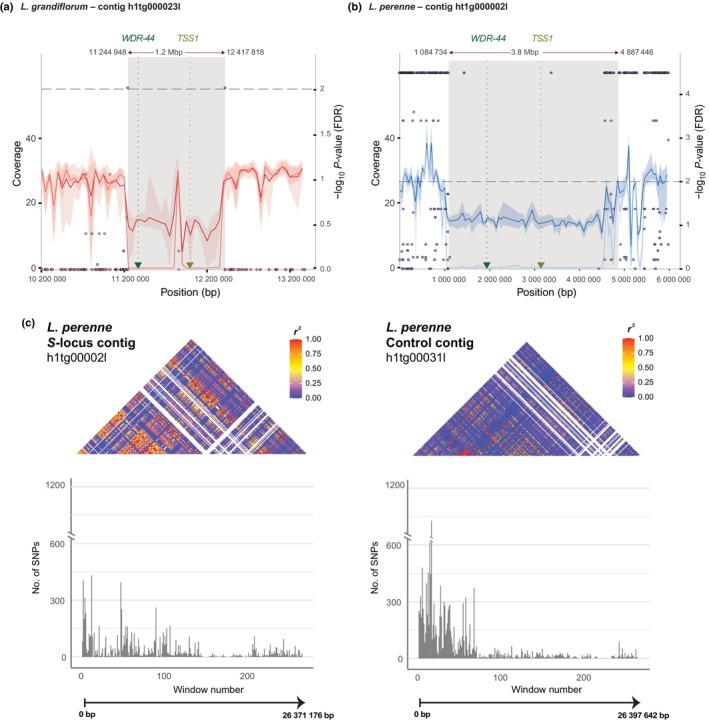
Identification and characterization of *S‐*loci and its genomic regions in *Linum grandiflorum* and *Linum perenne*. Both *L. grandiflorum* contig h1tg000023l (a) and *L. perenne* contig ht1g000002l (b) harbor S‐morph hemizygous *S‐*linked regions (coverage values, left y‐axis), which contain candidate distyly genes *TSS1* and *WDR‐44*. The size of the hemizygous region and the pattern of single‐nucleotide polymorphism (SNP) association (points showing genome‐wide association study (GWAS) significance vs position, right y‐axis, significance level α = 0.01 indicated by a dashed line) differ between species. In each plot, darker and lighter lines correspond to S‐morph and L‐morph normalized coverage, respectively, surrounded by shaded regions indicating 95% confidence intervals. The gray areas correspond to regions hemizygous in S‐morph individuals, based on coverage analysis and alignment of haplotype‐resolved assemblies. The positions of candidate genes *TSS1* and *WDR‐44* are indicated by dotted lines and arrows and the x‐axis shows position on each contig (in base pairs). (c) Extended linkage disequilibrium in a *L. perenne* natural population along *S*‐locus contig h1tg000002l compared to control contig ht1g00031l. Heatmaps of median *r* ^2^ values between all pairs of windows of 100 kb as well as the number of SNPs along both contigs are shown. White areas in the heatmap represent the lack of SNPs. The black lines and arrows represent the lengths of the contigs. linkage disequilibrium (LD) distributions along the two contigs were significantly different (Wilcoxon rank sum test, W = 600 311 350, *P*‐value < 0.001).

In *L. grandiflorum*, GWAS identified two SNPs on contig h1tg000023l of haplotype‐resolved assembly hap1 as significantly associated with floral morph (Fisher exact test, assuming dominant effect of the S‐morph‐specific allele, FDR < 0.05; Figs 2a, S1). The associated SNPs define an *c*. 1.2‐Mb region on contig h1tg000023l ranging from *c*. 11.2 Mb to *c*. 12.4 Mb. Within this region, coverage analyses showed presence–absence variation between floral morphs, with significantly lower normalized median coverage in L‐morph than S‐morph individuals (median normalized coverage 0 for L‐morph and 14.8 for S‐morph, permutation test with 1000 000 resamples, Bonferroni‐corrected *P* < 0.01, Fig. 2a). These results suggest that h1tg000023l on haplotype‐resolved assembly hap1 harbors the longer, dominant allele at the *L. grandiflorum S‐*locus. Comparison between the two haplotype‐resolved assemblies of *L. grandiflorum* confirmed the presence of a *c*. 1.2‐Mb hemizygous region in S‐morph individuals and identified h2tg000012l on the hap2 assembly as harboring the shorter, recessive *S‐*allele. Although the recessive allele was shorter than the dominant allele, it included a unique 70‐kb region missing from the dominant allele. Finally, inspection of the *L. grandiflorum* genome annotation showed that the S‐morph‐specific region on the dominant *S‐*allele (i.e. on h1tg000023l) harbored the *S‐*linked gene *TSS1* (Ushijima *et al*., 2015; Figs 2a, S1). Taken together, these results indicate that the *S‐*locus of *L. grandiflorum* includes a *c*. 1.2‐Mb genomic region which is hemizygous in S‐morph individuals.

In *L. perenne*, family‐based GWAS analysis resulted in significant associations between floral morph and SNP genotype on four contigs. We identified a total of 124 significantly associated SNPs on contig ht1g000002l, 33 on contig ht1g000009l, 2 on ht1g000026l and 20 on ht1g000047l of our hap1 haplotype‐resolved genome assembly (Fisher exact test, assuming dominant effect of the S‐morph‐specific allele, FDR < 0.01; Figs 2b, S1). These results were validated using a population‐based GWAS which showed that the same four contigs accounted for 97.7% of GWAS hits (Notes S2). Both contigs with the highest number of morph‐associated SNPs (ht1g000002l and ht1g000009l on hap1) map to the same contig in the alternate haplotype‐resolved genome assembly (ht2g000035l on hap2), implying that at least 87.7% of the SNPs that show an association with floral morph map to the same chromosome.

Coverage analyses further indicate that hap1 contig ht1g000002l corresponds to the dominant *S‐*allele, as it harbored an *c*. 3.8‐Mb region specific to the S‐morph (median normalized coverage 14.7 in S‐morph, 0 in L‐morph; Fig. 2b), and additionally identified an *c*. 800‐kb region specific to the recessive allele (hemizygous in S‐morph and diploid in L‐morph). Inspection of the annotation of the *S‐*haplotype showed the presence of an ortholog of *TSS1* (Figs 2b, S1; Table 1; Dataset S1B). The *L. perenne S‐*locus thus includes an *c*. 3.8‐Mb region that is specific to and hemizygous in the S‐morph (Figs 2b, S1).

**Table 1:** Total number of protein‐coding genes annotated at the S‐locus of *Linum grandiflorum*, *Linum perenne* and *Linum tenue*, the number and identity of genes shared among S/s alleles and between species, and other identified genes of potential interest for floral morphology/distyly.

*S‐*haplotype	Species					
	*L. grandiflorum*		*L. perenne*		*L. tenue*	
	*S*‐Dominant^1^	*s*‐Recessive^1^	*S*‐Dominant^1^	*s*‐Recessive^1^	*S*‐Dominant^1^	*s*‐Recessive^1^
Total gene count^2^	24	4 (2)^3^	32	11 (3)^3^	9	2 (2)^3^
Shared gene count	3 (2)^4^	0	3 (2)^4^	0	2^4^	0
Shared genes	*TSS1* ^4^, *WDR‐44* ^4^, *MPT1* ^5^	–	*TSS1* ^4^, *WDR‐44* ^4^, *MPT1* ^5^	–	*TSS1* ^4^, *WDR‐44* ^4^	–
Other genes of interest	*NOV*	*NOV*	*AGL8*, *AGL80*	*AGL80*		

^1^ Separate gene counts are given for the dominant (*S*) and recessive (*s*) *S‐*haplotypes of each species.

^2^ Gene counts exclude genes with transposable element‐related functional annotation. For detailed annotation information on listed genes, see Dataset S1B.

^3^ Counts of annotated genes on the recessive haplotype that were also present on the dominant haplotype given in parentheses.

^4^ Annotated genes shared between all three species.

^5^ Annotated genes shared between *L. grandiflorum* and *L. perenne* only.

Taken together, association mapping, coverage analyses, and comparisons between haplotype‐resolved assemblies indicate that, like in *L. tenue*, the distyly *S‐*loci of *L. grandiflorum* and *L. perenne* each contain a region present only in the *S*‐allele. This S‐morph‐specific hemizygous region is considerably larger in *L. grandiflorum* (*c*. 1.2 Mb) and *L. perenne* (*c*. 3.8 Mb) than in *L. tenue* (*c*. 260 kb; Gutiérrez‐Valencia *et al*., 2022).

### Extended linkage disequilibrium around the *S*‐morph hemizygous region in *L. perenne*

In total, the contigs showing an association between floral morph and SNP genotype span > 30 Mb in *L. perenne*. The large size of the region associated with floral morph is likely due to extended recombination suppression, which is demonstrated by elevated LD in this specific genomic region (Fig. 2c), in three natural populations (Notes S3; Fig. S2). This suggests that the genomic region with limited recombination is longer in *L. perenne* than in *L. grandiflorum*, where GWAS hits were in the immediate vicinity of the S‐hemizygous region (Figs 2a,b, S1a–d). Similarly, no extended LD was detected beyond the S‐hemizygous region in *L. tenue* (Gutiérrez‐Valencia *et al*., 2022).

### Divergent *S*‐loci are enriched for different classes of repeats

**Fig. 3:**
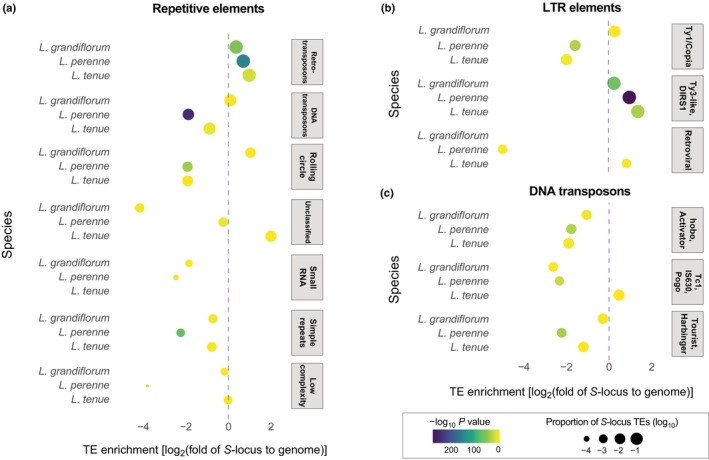
Repetitive element content of the hemizygous *S‐*locus regions of *Linum grandiflorum* and *Linum perenne*, compared to *Linum tenue*. The log_2_‐fold enrichment of repetitive elements (a), long terminal repeat (LTR) elements (b) and DNA transposons (c) at the S‐morph hemizygous *S‐*locus region of *L. grandiflorum*, *L. perenne* and *L. tenue*. Colors indicate the −log10 *P*‐value from a binomial test of repeat enrichment. Circle sizes denote the (log_10_‐transformed) proportion of the *S‐*locus region made up of a certain type of repeat.

The *S‐*locus is expected to accumulate repeats due to the combined effects of lack of recombination and reduced effective population size (reviewed by Gutiérrez‐Valencia *et al*., 2021a, 2021b). In line with this expectation, we found that the *S‐*locus was enriched in repetitive elements relative to the genome‐wide average in both *L. perenne*, *L. grandiflorum* and *L. tenue* (Fig. 3a; Table S3). Enrichment was driven primarily by retroelements, specifically Ty3‐like long terminal repeat (LTR) retroelements (Fig. 3b; Table S3). Consistent with the independent accumulation of repeats in different lineages after an early cessation of recombination, the content of certain classes of TEs differed between species, with rolling circle TEs overrepresented at the *S‐*locus of *L. grandiflorum* (Fig. 3c).

### Divergent *S*‐loci share distyly candidate genes despite pervasive differences in gene content

**Fig. 4:**
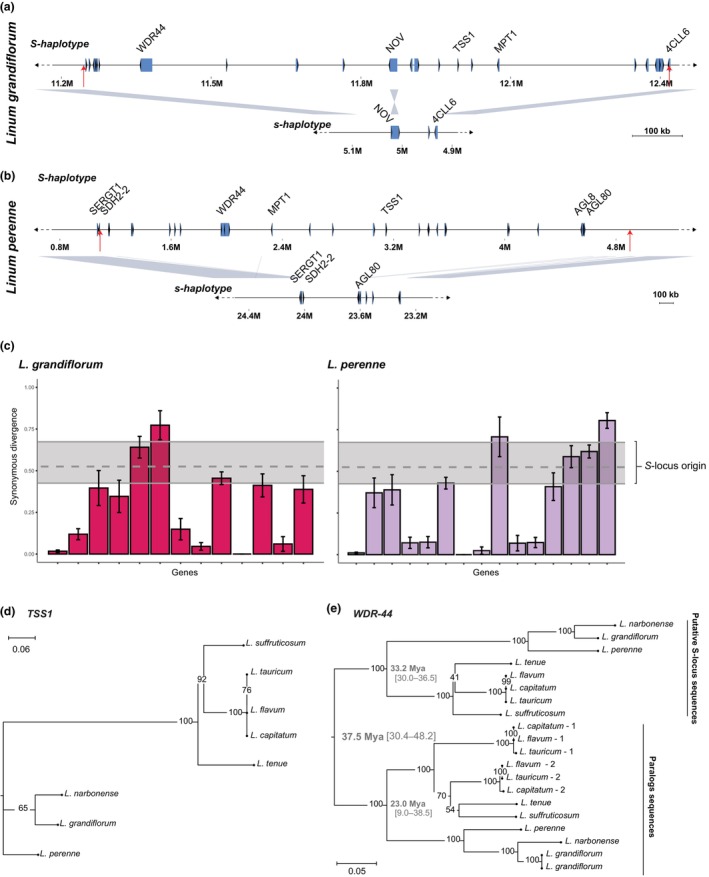
Gene content, evidence for stepwise assembly of the hemizygous *S‐*locus regions and conserved S‐locus genes in *Linum grandiflorum* and *Linum perenne*. (a, b) Schematic depiction of the haplotype structure and gene content on the dominant and recessive alleles at the hemizygous region of the *L. grandiflorum* (a) and *L. perenne* (b) *S‐*locus. Genes are indicated by blue boxes and arrows indicating orientation. Names of candidate genes (*S‐*linked genes shared between *Linum* species) and genes present on both the recessive (*s*) and dominant (*S*) haplotypes are shown. Red vertical arrows indicate S‐hemizygous region boundaries. (c) Synonymous divergence between *S*‐locus genes and their closest paralogs in *L. grandiflorum* and *L. perenne*. Variation in synonymous divergence values between the different *S*‐locus genes and their paralogs across both species supports a stepwise formation of the gene set at the *S*‐locus. The black dashed line represents the divergence time between *WDR‐44* and its paralogs, while the black solid lines indicate the 95% confidence interval (CI) for this divergence time. SE bars are shown. For some genes, the synonymous divergence is lower than the estimated emergence time of the *S*‐locus in *Linum*, suggesting that these genes were incorporated into the *S*‐locus after its initial formation. (d) Phylogenetic tree of the conserved *S‐*locus candidate genes *TSS1* for style polymorphic *Linum* (all distylous except *L. grandiflorum* which exhibits stigma height dimorphism) reconstructed using RAxML under a GTR‐GAMMA substitution model. Support values based on 100 bootstraps are indicated by each node. (e) Phylogenetic tree of the conserved *S‐*locus candidate genes *WDR44* for style length polymorphic *Linum* reconstructed using RAxML under a GTR‐GAMMA substitution model. Support values based on 100 bootstraps are indicated by each node. Estimates of the inferred timing of duplication and age of each clade based on BEAST2 analysis are shown for *WDR‐44*, with 95% CIs indicated in square brackets.

We compared the gene content of the *S‐*loci of *L. perenne*, *L. grandiflorum*, and *L. tenue* and found that only *TSS1* and *WDR‐44* were *S‐*linked in all species. This finding suggests that *TSS1* and *WDR‐44* were *S‐*linked in the most recent common ancestor of these species *c*. 33 Ma. One additional *S*‐linked gene, *MPT1* (Mitochondrial Phosphate Transporter; GO:0005315) was present in both *L. perenne* and *L. grandiflorum* (Fig. 4a,b). No other gene homology was detected when comparing the gene content of the hemizygous region of *L. grandiflorum* to the four morph‐associated contigs of *L. perenne* (Figs 4a,b, S3; Table 1; Notes S4). The number of annotated genes in the *S‐*linked hemizygous region differed greatly between the three species, with the *S*‐haplotype having 24 vs 32 annotated protein‐coding genes in *L. grandiflorum* and *L. perenne*, compared with only nine in *L. tenue* (Gutiérrez‐Valencia *et al*., 2022; Table 1, Dataset S1B).

In *Primula* (Huu *et al*., 2020) and *L. tenue* (Gutiérrez‐Valencia *et al*., 2022), the *S*‐locus gene set was likely assembled in a stepwise fashion through gene duplication. Differences in gene content between *Linum S*‐loci could result from continued gene duplication and/or gene loss at the *S*‐locus region (Notes S4). To test for the former, we estimated *d*_*S*_ between *S‐*locus genes and their closest paralogs in *L. grandiflorum*, *L. perenne* and *L. tenue*. The results revealed wide variation in the timing of duplication, including very recent duplication (Figs 4c, S3). Additionally, these paralogs were found on multiple contigs in both *L. grandiflorum* and *L. perenne* (Table S4). Together, these results suggest that stepwise gene duplication, as well as gene loss, occurring independently in diverged *Linum* lineages, has contributed to the differences in S‐locus gene content we observe.

### Functional constraints on distyly candidate genes at the *S*‐locus over 30 Ma

The presence of *TSS1* and *WDR‐44* on the *S*‐haplotypes of the *S‐*loci of *L. grandiflorum*, *L. perenne* and *L. tenue* (Gutiérrez‐Valencia *et al*., 2022) suggests that these genes were *S‐*linked already *c*. 33 Ma and conserved due to their function in the determination of floral morph and/or SI. A simple molecular clock analysis of synonymous divergence at *TSS1* and *WDR‐44* between *L. perenne* and *L. tenue* supports this conclusion, as it placed the split between these species at *c*. 31–37 Ma (*TSS1: t* = 36.6 Ma (±SE 7.3 Ma), *d*_*S*_ = 0.513 ± 0.10*; WDR‐44: t* = 31.3 Ma (±SE 2.1 Ma), *d*_*S*_ = 0.438 ± 0.03; Fig. 4d,e), consistent with the retention of these genes since the diversification of *Linum c*. 33 Ma.

While *TSS1* is a single‐copy gene with homologs in outgroups of *Linum*, *WDR‐44* is part of a gene family and harbors non‐*S‐*linked paralogs (six genes in total in *L. perenne* and *L. tenue* (Gutiérrez‐Valencia *et al*., 2022), five in *L. grandiflorum*). To determine when *TSS1* and *WDR‐44* first became co‐localized in the genome, and to quantify sequence‐level constraint on these distyly candidate genes, we assembled sequences of *TSS1*, *WDR‐44* and a set of paralogs of *WDR‐44* from five additional distylous *Linum* species (Figs 1a, 4d,e; Table S1). Phylogenetic analysis indicated that the *S*‐linked copy of *WDR‐44* originated by gene duplication *c*. 37 Ma (95% highest posterior density (HPD) interval: 30.4–48.2 Ma), suggesting that duplication and translocation of *WDR‐44* into a genomic region already harboring *TSS1* occurred at or before the diversification of *Linum* (estimated to have occurred 33 Ma (95% HPD: 27.2–38.3 Ma; Maguilla *et al*., 2021; Figs 4e, S4)). Consistent with this hypothesis, the closely related outgroup *T. sinensis* only harbored a sequence clustering with the non‐*S‐*linked paralogs of *WDR‐44*, while *WDR‐44* sequences of more distant outgroups fell outside the Linaceae. Taken together, these results support our inference that *WDR‐44* duplication occurred at or around the time of diversification of *Linum* (Fig. S4).

If *TSS1* and *WDR‐44* were retained at the distyly *S‐*locus in *Linum* for > 30 Ma, we would expect these genes to be under functional constraint. To test this hypothesis, we analyzed ratios of nonsynonymous to synonymous divergence (*d*_*N*_ */d*_*S*_) across our eight *Linum* species. We found that for both *TSS1* and the *S‐*linked copy of *WDR‐44*, a simple model with a single *d*_*N*_ */d*_*S*_ across our *Linum* species was supported (*TSS1*: LRT, log LRT test statistic: 1.93, df = 8, *NS*; *WDR‐44*: LRT, log LRT test statistic: 1.18, df = 8, *NS*), and both *d*_*N*_ */d*_*S*_ estimates were well below 1 (*d*_*N*_ */d*_*S*_ of 0.29 ± 0.05 for *TSS1*, and 0.37 ± 0.03 for *WDR‐44*), consistent with both genes being under purifying selection (see also Notes S5). However, the *S*‐locus copy of *WDR‐44* exhibited elevated *d*_*N*_ */d*_*S*_ compared with its paralogs (0.37 ± 0.03 vs 0.27 ± 0.02; LRT, log LRT test statistic = 9.08, df = 2, *P* = 0.0107). This could suggest either relaxed purifying selection or more frequent positive selection on the *S‐*locus copy of *WDR‐44*, which might be expected under a model of duplication and neofunctionalization associated with *S*‐locus formation. As we could not detect positive selection at the duplication node (LRT statistic = 0.11, df = 1, *P* = 0.74), we cannot reject relaxed purifying selection as a cause for the elevated *d*_*N*_ */d*_*S*_ of the *S*‐locus copy of *WDR‐44* relative to its paralogs.

These results suggest that the distyly *S‐*locus of *Linum* formed at or before the diversification of *Linum* and that the two *S*‐locus candidate genes *TSS1* and *WDR‐44*, which are shared among widely diverged distylous *Linum* species, are under purifying selection, possibly related to their role in determining floral morph differences and/or SI.

### Regulation of style length by brassinosteroids in widely divergent distylous *Linum*

The style length candidate gene *TSS1* was present and conserved at the *S‐*loci of the three species, which all exhibit style length polymorphism. We previously hypothesized that *TSS1*, primarily expressed in styles of S‐morph individuals (Ushijima *et al*., 2015; Gutiérrez‐Valencia *et al*., 2022), might result in shorter style cells and thereby shorter styles by downregulating brassinosteroid‐responsive genes in a manner similar to its *Arabidopsis* homolog *VUP1* (Grienenberger & Douglas, 2014). If so, treating floral buds with brassinosteroids should result in longer styles and style cells specifically in S‐morph but not in L‐morph *Linum* individuals. If the mechanism of action of *TSS1* has remained conserved, we expect the effect of brassinosteroid treatment to be present in widely divergent distylous *Linum* species, as long as their *S‐*locus harbors functional *TSS1*. To test this hypothesis, we conducted a brassinosteroid supplementation experiment where L‐ and S‐morph flower buds of *L. tenue* and *L. perenne* were treated with brassinosteroid solution (eBL: 10 μM 24‐eBL, dissolved in 0.1% of the solvent DMSO) or control treatment (control: 0.1% DMSO only), followed by measurement of style and stamen length.

**Fig. 5:**
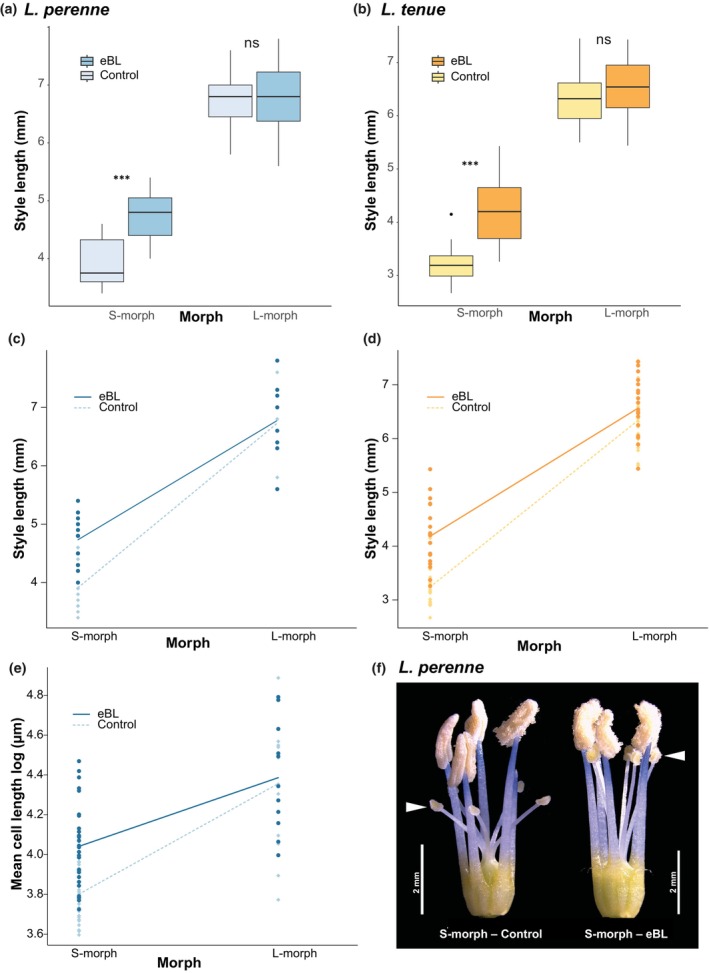
Brassinosteroid supplementation results in longer styles and style cells in S‐morph but not L‐morph morph individuals of widely divergent *Linum* species. (a, b) Boxplots showing significantly longer S‐morph but not L‐morph styles in *L. perenne* (a) and *L. tenue* (b) after epibrassinolide (10 μM epibrassinolide (eBL) in 0.1% dimethylsulfoxide (DMSO)) treatment of flower buds compared with control treatment (0.1% DMSO only). In the boxplots, the thick line represents the median, and the upper and lower limits of the box the third and first quartile, respectively. Whiskers extend to the outermost observation within 1.5 times the interquartile range. Significance values from a Tukey Honest Significant Difference (HSD) test are indicated in a and b, with ns indicating *P* ≥ 0.05) and ***, *P* < 0.001. (c, d) Interaction plots demonstrating a significant interaction between floral morph and eBL treatment, in both *L. perenne* (c) and *L. tenue* (d). (e) eBL treatment results in significantly longer epidermal style cells in S‐morph but not L‐morph individuals of *L. perenne*. (f) Photograph of control and eBL‐treated *L. perenne* S‐morph sexual organs, showing the effect of eBL treatment on style length. Stigma positions are indicated by arrows.

In line with our expectation, eBL treatment resulted in significantly longer styles in both *L. perenne* (Table 2; Fig. 5a) and *L. tenue* (Table 2; Fig. 5b) due to the specific effect of eBL treatment on style length in S‐morph individuals (*L. perenne*: two‐way ANOVA, interaction between treatment and morph; Table 2; Fig. 5c; *L. tenue*: two‐way ANOVA, interaction between treatment and morph; Table 2; Fig. 5d). On average, eBL treatment resulted in 0.82 mm (95% CI: 0.34–1.30 mm; Fig. 5f) longer styles in *L. perenne* S‐morph individuals and 0.94 mm (95% CI: 0.50–1.38 mm) longer styles in *L. tenue* S‐morph individuals. While styles were significantly longer in eBL‐treated S‐morph individuals, they were still shorter than those of L‐morph individuals (Fig. 5a), possibly due to the timing of application and/or concentration of eBL treatment used. The eBL treatment had no effect on style length in L‐morph individuals of *L. perenne* or *L. tenue* (Fig. 5a,b), and there was no significant interaction effect on stamen length in *L. perenne* or *L. tenue* (Table S5).

**Table 2:** Brassinosteroid treatment has a morph‐specific effect on style length (mm) in both the distylous species *Linum perenne* and *Linum tenue*.

Species	Source of variation^1^	Df^2^	SS^3^	MS^4^	F^5^	*P*‐value
*L. perenne*	Morph	1	60.36	60.36	244.24	< 0.0001
	Treatment	1	3.71	3.71	15.03	< 0.0001
	Morph*Treatment	1	1.51	1.51	6.11	0.02
	Residuals	42	10.38	0.25		
*L. tenue*	Morph	1	142.7	142.7	537.1	<0.0001
	Treatment	1	6.48	6.48	24.4	<0.0001
	Morph*Treatment	1	2.44	2.44	9.2	0.003
	Residuals	72	19.13	0.27		

^1^ Analysis of variation (ANOVA) sources of variation.

^2^ Degrees of freedom.

^3^ Sums of squares.

^4^ Mean squares.

^5^ F‐statistic.

To test whether the effect of brassinosteroid treatment on style length was mediated by style cell length, we measured epidermal style cell length in *L. perenne* after eBL and control treatment. There was a significant effect of eBL treatment on mean style cell length (*F*_*1*,*75*_, *P* < 0.0001; Figs 5e, S5a; Table S6), as well as a significant interaction between eBL treatment and morph (*F*_1,75_ = 5.7, *P* = 0.02; Fig. 5e; Table S6). The effect of eBL on mean style cell length in the S‐morph was 12.6 μm (95% CI: 4.9–20.3 μm). The brassinosteroid treatment had no significant effect on mean style cell length in L‐morph individuals (Figs 5e, S5).

Taken together, the impact of brassinosteroid treatment on style and style cell length specifically in S‐morph individuals suggests that a mechanism relying on the brassinosteroid pathway, likely regulated by *TSS1*, contributes to style length differences between floral morphs in widely diverged distylous *Linum*.

## Discussion

One of the most prominent examples of convergent floral evolution in plants is distyly, yet until recently little was known about the underlying mechanisms driving this multi‐trait balanced polymorphism and its mode of origin. Here, we leverage haplotype‐resolved genome assemblies of widely diverged *Linum* species to shed light on the molecular basis of distyly.

We show that the *S*‐locus supergenes of three *Linum* species that diverged as far back as 33 Ma all harbor an S‐morph‐specific hemizygous region. All three species share only two genes at the S‐morph‐specific region of their *S‐*loci: the style length candidate gene *TSS1* (Ushijima *et al*., 2015; Gutiérrez‐Valencia *et al*., 2022) and *WDR‐44*, hypothesized to control anther height and/or pollen SI (Gutiérrez‐Valencia *et al*., 2022, 2024). We have previously shown that *TSS1* is present in outgroups of *Linum* (Gutiérrez‐Valencia *et al*., 2022), and here, we used paralog dating to show that *WDR‐44* originated through gene duplication *c*. 37 Ma (95% CI: 30.4–48.2 Ma), suggesting that these two distyly candidate genes became colocated in one genomic region at or before the diversification of *Linum* s.l. *c*. 33 Ma (Maguilla *et al*., 2021). The distyly *S‐*locus therefore probably evolved early during the diversification of *Linum*, through a process involving duplication of *WDR‐44*, as previously documented for the anther height gene *GLO2* (*GLO* ^*T*^) at the distyly supergene in *Primula* (Li *et al*., 2016; Huu *et al*., 2020). *TSS1* was already present and could have evolved presence–absence polymorphism regulating style length before the duplication of *WDR‐44* and its co‐location with *TSS1*, broadly in line with predictions of the ‘pollen transfer’ model of the evolution of distyly (Lloyd & Webb, 1992). However, we cannot currently rule out other scenarios (Charlesworth & Charlesworth, 1979), including one where both stamen and style length polymorphism were established at the same time through a large indel generating presence–absence variation for both *TSS1* and *WDR‐44*. Additionally, we cannot currently rule out that other genes were originally present but were lost in the lineages leading to *L. tenue* and both *L. perenne* and *L. grandiflorum*.

Molecular evolutionary analyses showed that *TSS1* and *WDR‐44* are under purifying selection in *Linum*. The presence of *WDR‐44* at the *S‐*locus of *L. grandiflorum* is intriguing as this species lacks stamen length polymorphism. On the contrary, *L. grandiflorum* shares heteromorphic SI with both *L. tenue* and *L. perenne* (Murray, 1986), so we cannot rule out conservation of *WDR‐44* in *L. grandiflorum* due to an effect of this gene on pollen SI. Further detailed characterization and functional work will be required to determine the effects of *WDR‐44* on distyly in *Linum*. Regarding *TSS1*, it has been hypothesized to regulate style length via its impact on the brassinosteroid pathway (Gutiérrez‐Valencia *et al*., 2022). While functional studies of *TSS1* are required to validate our findings, morph‐specific effects of exogenous brassinosteroid application on style and style cell length in *L. perenne* suggest that *TSS1* governs style length through its effect on brassinosteroid‐regulated genes, highlighting that sequence conservation at this gene is likely related to its effect on distyly. Furthermore, brassinosteroid supplementation had a morph‐specific effect on style length in both *L. perenne* and *L. tenue*, which implies that the mechanism underlying style length polymorphism is conserved across these widely diverged distylous *Linum* species. Future studies should investigate whether brassinosteroid treatment also affects female SI reaction in *Linum*. While we cannot fully rule out the involvement of additional hormonal pathways in the regulation of style length in *Linum*, our results suggest that genes impacting the brassinosteroid pathway have repeatedly been recruited during the convergent evolution of style length polymorphism in distylous species, including in *Primula* (Huu *et al*., 2022) and *Turnera* (Matzke *et al*., 2020, 2021).

Besides these shared features across all three species, our study also revealed strong differences in overall gene content at the *S‐*locus across divergent *Linum* species. While denser sampling of high‐quality genome assemblies will be required to infer how gene loss has shaped *S‐*locus gene content, recent genomic studies have documented gene movement to the *S‐*locus (Huu *et al*., 2020; Gutiérrez‐Valencia *et al*., 2022). Consistently, we found evidence for stepwise gene movement to the *S‐*locus, occurring independently and continuously in the studied *Linum* species. High repeat content at the *S‐*locus in combination with reduced selection against structural variation in the nonrecombining hemizygous region could have facilitated this process. This abundance of repetitive elements, particularly retrotransposon and rolling circle TEs, such as Helitrons, which have been demonstrated to mobilize host genes in maize (Lai *et al*., 2005; Yang & Bennetzen, 2009), may have promoted gene transposition to the *S‐*locus via ectopic recombination or gene capture. Additionally, in a process resembling the theoretical accumulation of sexually antagonistic genes in sex‐determining regions (Rice, 1987; Otto, 2014), the difference in gene content between these species could further be the result of lineage‐independent accumulation of thrum morph favored genes on the thrum‐specific *S‐*haplotype, even if they were pin‐detrimental.

This study also reveals large variation in the size of the hemizygous region (from *c*. 260 kb to *c*. 3.8 Mb) as well as variation in the extent of recombination suppression at the *S*‐locus region. The hemizygous *S*‐locus region likely independently expanded over time after an initial early cessation of recombination, consistent with the observed differences in repeat content in the three species. Additional studies should further characterize the recombination landscape at this genomic region in *L. perenne* and test whether extensive recombination suppression could be the result of evolutionary processes analogous to those at sex‐determining regions (reviewed by Charlesworth, 2016) and whether discrete stepwise extension, similar to the evolutionary strata of sex chromosomes, could have occurred.

Along with recombination suppression between alleles and morph‐specific inheritance of one allele (reviewed in Gutiérrez‐Valencia *et al*., 2021b), additional analogies can be found between distyly supergenes and plant sex‐determining regions. For instance, plant sex‐determining loci often harbor regions specific to the heterogametic morph (e.g. Y‐specific or W‐specific; Akagi *et al*., 2014, 2023; Tennessen *et al*., 2018; Scharmann *et al*., 2019; Harkess *et al*., 2020; Müller *et al*., 2020), are often enriched in TEs (Na *et al*., 2014; Akagi *et al*., 2023; Sacchi *et al*., 2024), can exhibit rampant evolution of gene content, and undergo turnovers (Martin *et al*., 2019; Wang *et al*., 2022, 2024a, 2024b; Akagi *et al*., 2023; He *et al*., 2024; Sacchi *et al*., 2024). Most interestingly, many of the patterns documented here closely resemble those at the sex‐determining region of *Actinidia* species (Akagi *et al*., 2023). We observed only two shared candidate genes for distyly at the *S‐*haplotype of divergent *Linum* species, and Akagi *et al*. (2023) similarly observed only three shared candidate sex‐determination genes located in a male‐specific genomic region in divergent *Actinidia* species. Like Akagi *et al*. (2023), we also find that molecular mechanisms underlying morphs appear conserved, despite marked sequence‐level evolution of the morph‐specific region. These similarities suggest that further investigation of the parallels between the evolution of plant mating system supergenes and sex‐determining regions is a fruitful avenue for future theoretical and empirical work.

Taken together, our results shed light on the genetic architecture, origin and evolution of the *Linum* distyly supergene, revealing the presence of conserved candidate genes and pathways regulating distyly, despite marked differences in supergene size and recombination suppression, gene and repeat content. Especially, we assessed the timing and potential mode of origin of the S‐locus supergene in *Linum*. Our results and the genome assemblies produced here provide a foundation for further work on the role of parallel genetic changes for convergent evolution of floral form and function in distylous species.

## Competing interests

None declared.

## Author contributions

TS conceived of and designed the study, acquired funding, supervised the work and wrote the original draft. AL performed experiments. PIZ, ZP, MF, LS, EPW, IB, AC and TS performed analyses. PIZ, ZP and AL revised and edited the manuscript, with additional comments and input from MF, LS, EPW, IB and AC. PIZ and ZP contributed equally to this work.

## Disclaimer

The New Phytologist Foundation remains neutral with regard to jurisdictional claims in maps and in any institutional affiliations.

## Supporting information


**Dataset S1** Detailed annotation statistics and lists of S‐hemizygous genes.

**Fig. S1** *S‐*loci of *Linum grandiflorum* and *Linum perenne* harbor large S‐morph hemizygous regions containing distyly candidate genes.
**Fig. S2** Extended linkage disequilibrium in natural populations of *Linum perenne* along *S*‐locus contig h1tg000002l.
**Fig. S3** Synonymous divergence between *S*‐locus genes and their closest paralogs in *Linum perenne*, *Linum grandiflorum* and *Linum tenue*.
**Fig. S4** Phylogenetic tree of *WDR‐44*.
**Fig. S5** Brassinosteroid treatment results in longer style cells in S‐morph individuals of *Linum perenne*.
**Notes S1** Assembly contamination screening and masking results.
**Notes S2** Population‐based validation of GWAS results.
**Notes S3** Extensive linkage disequilibrium at the *L. perenne* distyly *S*‐locus.
**Notes S4** Description of gene content at the *S*‐loci of *L. grandiflorum* and *L. perenne*.
**Notes S5** Comparative molecular evolutionary analysis of rates of evolution of *TSS1* and *WDR‐44*.
**Table S1** Origin and section classification of plant material used in this study.
**Table S2** Genome assembly statistics for Hifiasm Hi‐C integrated haplotype‐resolved assemblies.
**Table S3** Repetitive element proportion of the hemizygous S‐loci and enrichment compared to the total genome.
**Table S4** Synonymous divergence for all genes with paralogs at the dominant allele *S‐*hemizygous regions and their closest paralogs in *Linum grandiflorum* and *Linum perenne*.
**Table S5** Brassinosteroid treatment has no detectable morph‐specific effect on stamen length (mm) in *Linum perenne* or *Linum tenue*.
**Table S6** Analysis of log‐transformed mean cell length data from the epibrassinolide supplementation experiment shows that brassinosteroid treatment has a morph‐specific effect on style cell length (mm) in *Linum perenne*.
Please note: Wiley is not responsible for the content or functionality of any Supporting Information supplied by the authors. Any queries (other than missing material) should be directed to the *New Phytologist* Central Office.

## Data Availability

All sequencing data generated in this study have been uploaded to the European Nucleotide Archive (ENA, https://www.ebi.ac.uk/ena/browser/view/PRJEB88074) with accession no.: PRJEB88074. NBIS open‐source pipelines for genome annotation are available at: https://github.com/NBISweden/GAAS; https://github.com/NBISweden/AGAT; https://github.com/NBISweden/pipelines‐nextflow.
