# Supplementary material for: Genomic studies in Linum shed light on the evolution of the distyly supergene and the molecular basis of convergent floral evolution
Source: New Phytol. 2025 Jul 18;247(6):2964–81. doi: 10.1111/nph.70392 (PMC12371154; doi:10.1111/nph.70392)
Supplement: Supplementary file 2 — Fig. S1 S‐loci of Linum grandiflorum and Linum perenne harbor large S‐morph hemizygous regions containing distyly candidate genes. Fig. S2 Extended linkage disequilibrium in natural populations of Linum perenne along S‐locus contig h1tg000002l. Fig. S3 Synonymous divergence between S‐locus genes and their closest paralogs in Linum perenne, Linum grandiflorum and Linum tenue. Fig. S4 Phylogenetic tree of WDR‐44. Fig. S5 Brassinosteroid treatment results in longer style cells in S‐morph individuals of Linum perenne. Notes S1 Assembly contamination screening and masking results. Notes S2 Population‐based validation of GWAS results. Notes S3 Extensive linkage disequilibrium at the L. perenne distyly S‐locus. Notes S4 Description of gene content at the S‐loci of L. grandiflorum and L. perenne. Notes S5 Comparative molecular evolutionary analysis of rates of evolution of TSS1 and WDR‐44. Table S1 Origin and section classification of plant material used in this study. Table S2 Genome assembly statistics for Hifiasm Hi‐C integrated haplotype‐resolved assemblies. Table S3 Repetitive element proportion of the hemizygous S‐loci and enrichment compared to the total genome. Table S4 Synonymous divergence for all genes with paralogs at the dominant allele S‐hemizygous regions and their closest paralogs in Linum grandiflorum and Linum perenne. Table S5 Brassinosteroid treatment has no detectable morph‐specific effect on stamen length (mm) in Linum perenne or Linum tenue. Table S6 Analysis of log‐transformed mean cell length data from the epibrassinolide supplementation experiment shows that brassinosteroid treatment has a morph‐specific effect on style cell length (mm) in Linum perenne. Please note: Wiley is not responsible for the content or functionality of any Supporting Information supplied by the authors. Any queries (other than missing material) should be directed to the New Phytologist Central Office. [file NPH-247-2964-s001.pdf]

New Phytologist

New Phytologist Supporting Information

Article title: **Genomic studies in _Linum_ shed light on the evolution of the distyly supergene and the molecular basis of convergent floral evolution**
Authors: Panagiotis-Ioannis Zervakis, Zoé Postel, Aleksandra Losvik, Marco Fracassetti, Lucile Solér, Estelle Proux-Wéra, Ignas Bunikis, Allison Churcher, Tanja Slotte
Article acceptance date: 24 June 2025

The following Supporting Information is available for this article:

Fig. S1 *S*-loci of *Linum grandiflorum* and *Linum perenne* harbor large S-morph hemizygous regions containing distyly candidate genes.
Fig. S2 Extended linkage disequilibrium in natural populations of *Linum perenne* along *S*-locus contig h1tg000002l.
Fig. S3 Synonymous divergence between *S*-locus genes and their closest paralogs in *Linum grandiflorum*, *Linum perenne*, and *Linum tenue*.
Fig. S4 Phylogenetic tree of *WDR-44*.
Fig. S5 Brassinosteroid treatment results in longer style cells in S-morph individuals of *Linum perenne*.
Table S1 Origin and section classification of plant material used in this study.
Table S2 Genome assembly statistics for Hifiasm Hi-C integrated haplotype-resolved assemblies.
Table S3 Repetitive element proportion of the hemizygous S-loci and enrichment compared to the total genome.
Table S4 Synonymous divergence for all genes with paralogs at the dominant allele *S*-hemizygous regions and their closest paralogs, in *Linum grandiflorum* and *Linum perenne*.
Table S5 Brassinosteroid treatment has no detectable morph-specific effect on stamen length (mm) in *Linum perenne* or *Linum tenue*.
Table S6 Analysis of log-transformed mean cell length data from the epibrassinolide supplementation experiment shows that brassinosteroid treatment has a morph-specific effect on style cell length (mm) in *Linum perenne*.
Note S1. Assembly contamination screening and masking results.
Note S2. Population-based validation of GWAS results.
Note S3. Extensive linkage disequilibrium at the *L. perenne* distyly S-locus.
Note S4. Description of gene content at the S-loci of *L. grandiflorum* and *L. perenne*.
Note S5. Comparative molecular evolutionary analysis of rates of evolution of *TSS1* and *WDR-44.*

Fig. S1 *S*-loci of *L. grandiflorum* and *L. perenne* harbor large S-morph hemizygous regions containing distyly candidate genes. Both *L. grandiflorum* (**a-b**) and *L. perenne* (**c-d**) harbor S-morph hemizygous *S*-linked regions (coverage values, left y-axis) which contain candidate distyly genes *TSS1* and *WDR-44*. The size of the hemizygous region and the pattern of SNP association (points showing GWAS significance vs position, right y-axis, significance level α=0.01 indicated by a dashed line) differ between species, with morph-associated SNPs found across the entire *S*-locus contig h1tg000002l in *L. perenne*. In each plot, darker and lighter lines correspond to S-morph and L-morph depth of coverage, respectively, surrounded by shaded regions indicating 95% confidence intervals. The grey areas correspond to regions hemizygous in S-morph individuals, based on coverage analysis and alignment of haplotype-resolved assemblies. The positions of candidate genes *TSS1* and *WDR-44* are indicated by dotted lines and arrows.

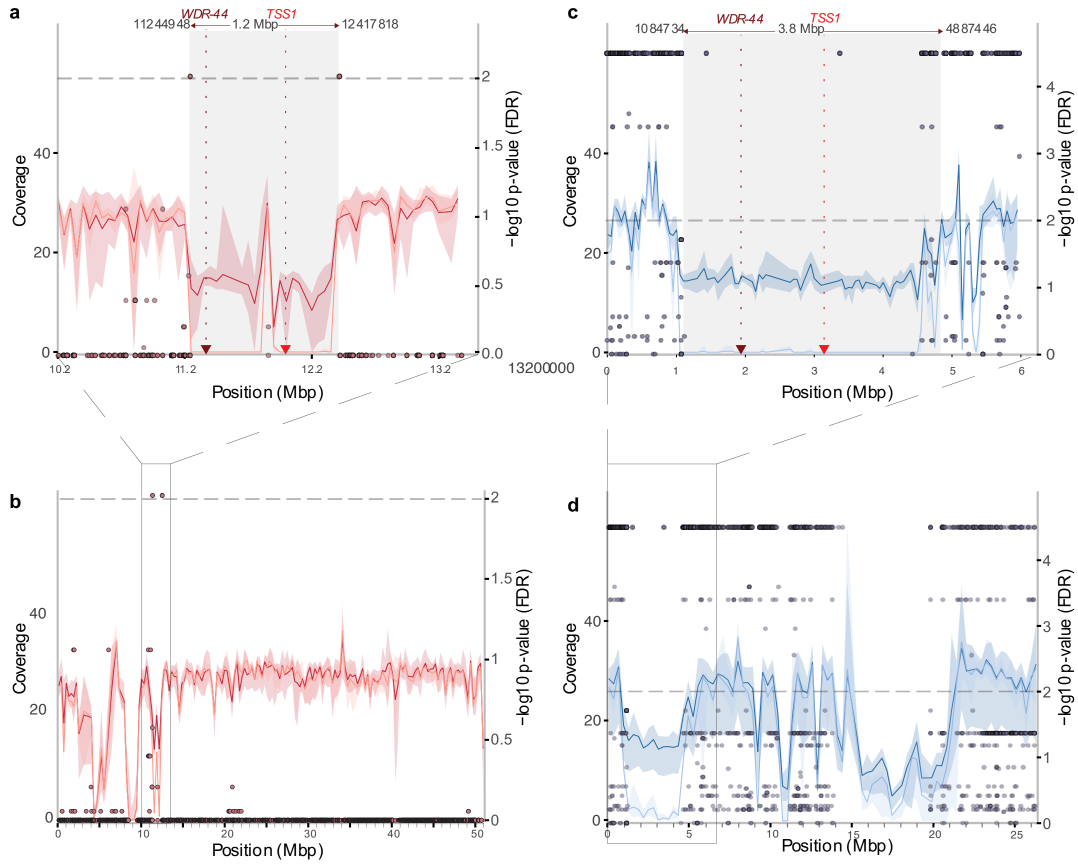

Fig. S2 Extended linkage disequilibrium in natural populations of *L. perenne* along *S*-locus contig h1tg000002l. Heatmaps of median r^2 values between all pairs of windows of 100 kb along the *S*-locus contig ht1g000002l and the control contig ht1g00031l for three natural populations of *L. perenne* (panels a-c). White areas in heatmap represent lack of SNPs. The lower plots show SNP number per window along each contig. LD distributions along the two contigs were significantly different for the two populations (Wilcoxon rank sum test, ger3: W = 355251530, p-value<0.001, ger5: W = 631400752, p-value<0.001, ger2: W = 600311350, p-value<0.001).

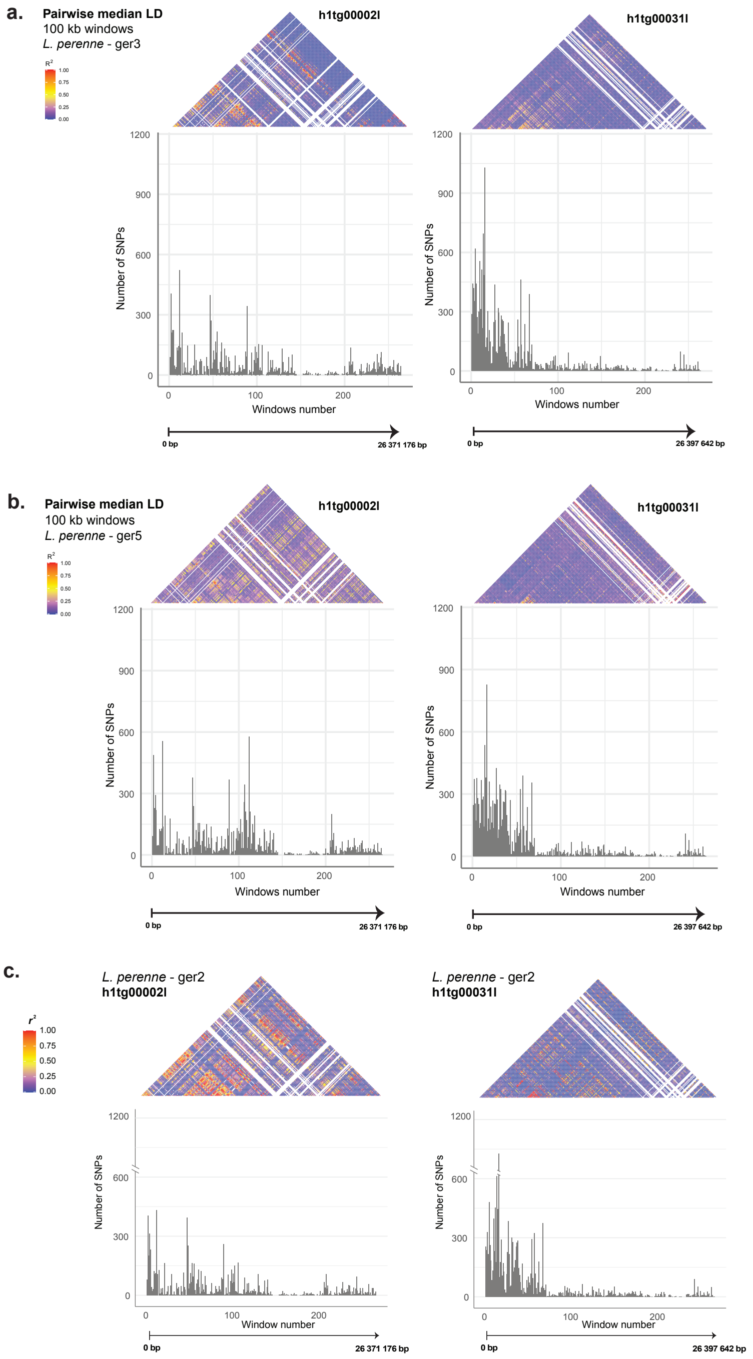

Fig. S3 Synonymous divergence between *S*-locus genes and their closest paralogs in *L. perenne*, *L. grandiflorum* and *L. tenue*. Variation in synonymous divergence values between the different S-locus genes and their paralogs across both species supports a stepwise formation of the gene set at the *S*-locus. The black dashed line represents the divergence time between *WDR-44* and its paralogs, while the black solid lines indicate the 95% confidence interval for this divergence time. For some genes, the synonymous divergence is lower than the estimated emergence time of the *S*-locus in *Linum*, suggesting that these genes were incorporated into the *S*-locus after its initial formation.

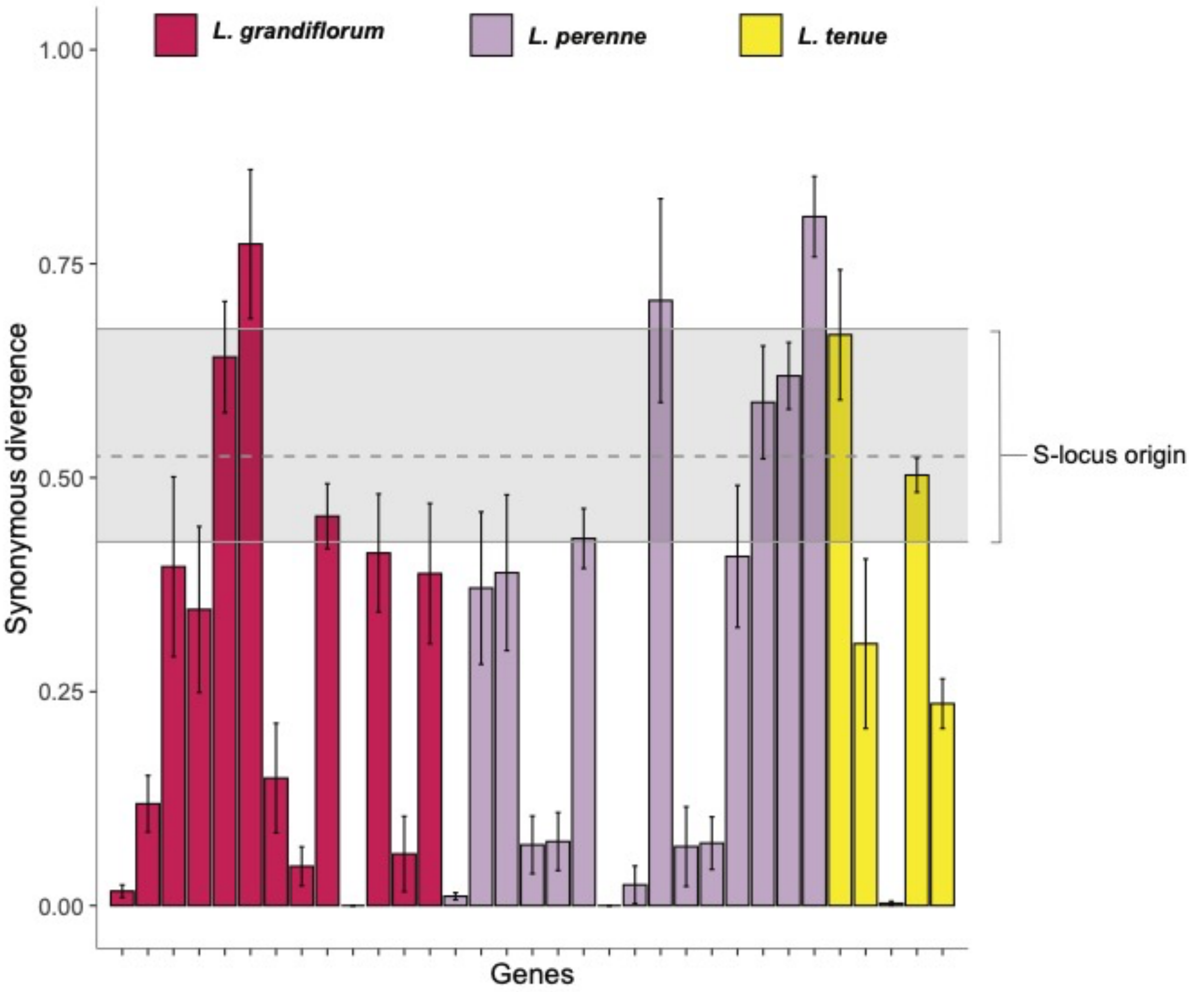

Fig. S4 Phylogenetic tree of *WDR-44*. This tree was reconstructed with RAxML and contains *S*-linked *WDR-44* sequences, paralog sequences and the sequence for one closely related outgroup (*Tirpitzia sinensis*) and two more distant outgroups (*Manihot esculenta* and *Populus trichocarpa*, respectively). The *Tirpitzia* sequence falls within the paralog cluster, supporting the emergence of the *S*-locus copy at the time of diversification of *Linum*.

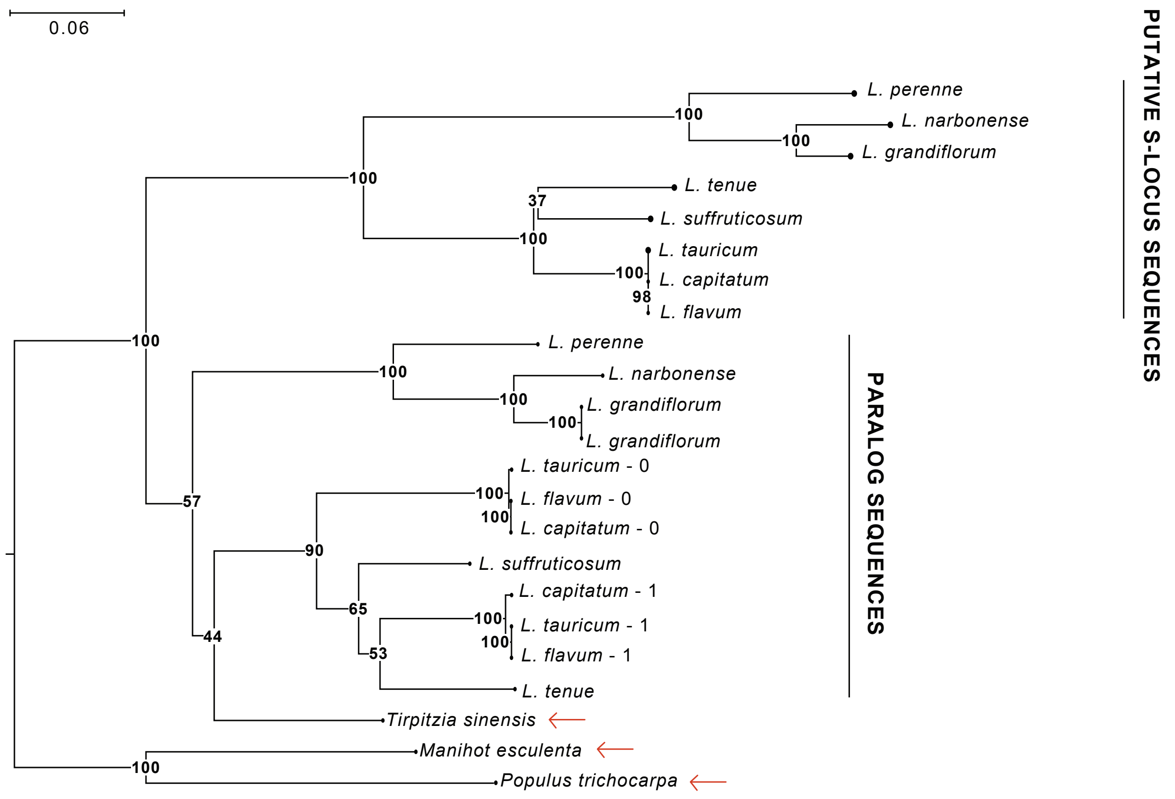

Fig. S5 Brassinosteroid treatment results in longer style cells in S-morph individuals of *L. perenne*. a. Effects of epibrassinolide (eBL) treatment on mean style cell length (μm) across all style sections. Mean style cell length was significantly longer after eBL treatment in S-morph (thrum) but not L-morph (pin) individuals. *** indicates P-value<0.001. NS indicates not significant. **b-d.** Boxplots showing mean style cell length with eBL or control treatment separately for each style section. **e.** Photograph indicating the three style sections where cells were measured, separately for an S-morph plant (left) and an L-morph plant (right). **f.** Example of micrograph of style cells after control (left) and eBL (right) treatment of an S-morph (thrum) plant.

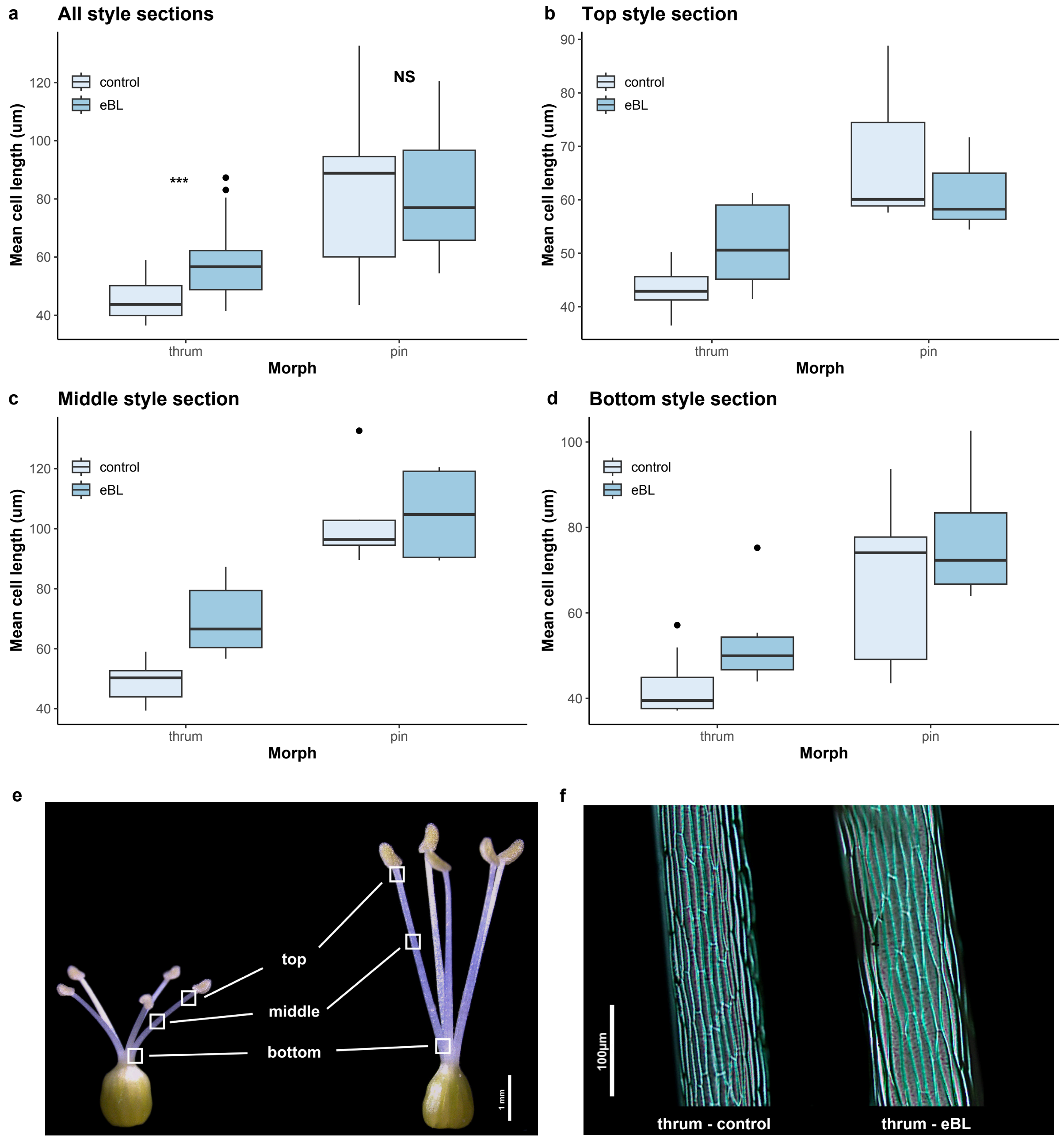

Table S1 Origin and section classification of plant material used in this study.

Species	Accession/origin	Section
Linum capitatum	LIN 1903 (IPK)	Syllinum
Linum flavum	LIN 2001 (IPK)	Syllinum
Linum grandiflorum	LIN 1389 (IPK)	Linum
Linum grandiflorum	LIN 974 (IPK)	Linum
Linum grandiflorum	LIN 10 (IPK)	Linum
Linum narbonense	LIN 1807 (IPK)	Linum
Linum perenne	LIN 2003 (IPK)	Linum
Linum perenne	ger2 near Othfresen, Germany	Linum
Linum perenne	ger3 near Marienhagen, Germany	Linum
Linum perenne	ger5 near Goslar, Germany	Linum
Linum suffruticosum	18-5-2 near Ronda, Spain	Linopsis C
Linum tauricum	LIN 1658 (IPK)	Syllinum
Linum tenue	CL, near Castillo de Locubín, Spain	Linopsis B
Linum tenue	SMT, near Santa María de Trassiera, Spain	Linopsis B

[1] Accessions labeled IPK are derived from the IPK Gatersleben Gene Bank and the IPK accession code is given in Accession/origin.

Table S2 Genome assembly statistics for Hifiasm Hi-C integrated haplotype-resolved assemblies.

	L. perenne assemblies			*L. grandiflorum* assemblies		
**Statistic**	**hap1**	**hap2**	**primary**	**hap1**	**hap2**	**primary**
**No. of contigs**	905	514	809	856	436	647
**Total length[1]**	739,225,655	753,366,752	773,825,471	816,779,520	743,719,982	809,872,110
**Largest contig**	41,207,707	49,950,927	87,425,665	90,933,564	60,021,352	125,429,925
**GC (%)**	41.45	41.67	41.46	42	41.81	41.86
**N50[2]**	10,271,661	11,313,697	23,398,707	28,074,137	22,188,264	69,915,768
**N75[3]**	4,808,757	5,584,177	14,389,690	15,858,823	12,991,397	38,093,872
**L50[4]**	19	17	10	9	12	5
**L75[5]**	44	41	21	18	22	8
**BUSCO**	95.1	94.8	94.7	94.4	94	94.4

[1]Flow-cytometry based genome size estimates for *L. perenne* and *L. grandiflorum* are 784 vs 908 Mb.

[2]N50: 50% of contigs in the assembly are longer than the N50 length.

[3]N75: 75% of the contigs in the assembly are longer than the N75 length.

[4]L50 Count of the smallest number of contigs which together make up 50% of the genome assembly size.

[5]L75: Count of the smallest number of contigs which together make up 75% of the genome assembly size.

Table S3 Repetitive element proportion of the hemizygous S-loci and enrichment compared to the total genome.

	Species											
	Linum grandiflorum				*Linum perenne*				*Linum tenue*			
**TE classification**	S-hemizygous Proportion [1]	Genome Proportion [1]	Enrichment (log2) [2]	p-value [3]	S-hemizygous Proportion [1]	Genome Proportion [1]	Enrichment (log2) [2]	p-value [3]	S-hemizygous Proportion [1]	Genome Proportion [1]	Enrichment (log2) [2]	p-value [3]
**Retrotransposons**	0.842	0.687	0.292	2.60E-58	0.928	0.599	0.631	9.80E-151	0.698	0.373	0.906	2.10E-20
**Ty1/Copia**	0.058	0.048	0.294	2.00E-01	0.018	0.054	-1.555	9.90E-34	0.023	0.089	-1.961	4.90E-05
**Ty3-like, DIRS1**	0.745	0.620	0.267	2.80E-66	0.886	0.447	0.987	3.70E-272	0.632	0.241	1.39	1.60E-38
**Retroviral**	0	>0.001	-Inf	1.00E+00	0.002	0.061	-4.963	7.00E-01	0.002	0.001	0.85	3.00E-01
**DNA transposons**	0.076	0.075	0.038	6.00E-04	0.029	0.112	-1.932	5.10E-240	0.081	0.155	-0.938	1.90E-07
**hobo, Activator**	0.004	0.009	-1.035	4.20E-05	0.005	0.017	-1.75	5.70E-36	0.011	0.042	-1.874	2.50E-04
**Tc1, IS630, Pogo**	0.003	0.018	-2.6	3.60E-01	0.002	0.008	-2.306	4.40E-25	0.02	0.015	0.485	7.00E-01
**Tourist, Harbinger**	0.007	0.009	-0.272	5.50E-04	0.003	0.013	-2.207	7.10E-39	0.008	0.019	-1.181	3.30E-02
**Rolling circle**	0.008	0.004	0.99	9.00E-01	0.005	0.021	-1.962	6.30E-43	0.011	0.044	-1.959	1.10E-03
**Unclassified**	0.002	0.037	-4.206	9.00E-05	0.003	0.003	-0.287	2.50E-06	0.024	0.006	1.953	6.60E-03
**Small RNA**	0	0.001	-2.028	8.00E-04	0	0.001	-2.638	1.90E-05	0	>0.001	-Inf	2.70E-01
**Simple repeats**	0.002	0.003	-0.772	7.00E-06	0.001	0.004	-2.284	8.60E-76	0.003	0.005	-0.82	1.80E-03
**Low complexity**	0	0.001	-0.229	2.60E-01	0	0.001	-3.85	1.10E-19	0.001	0.001	-0.06	1.00E+00

[1]Proportion composed of each category of transposable element

[2]Enrichment is calculated as the $\log_2\left(\frac{\text{TE proportion in S-hemizygous region}}{\text{TE proportion in genome}}\right)$

[3]Binomial test p-value for comparing the proportions of TE content of S-hemizygous regions to that of the total genome

Table S4 Synonymous divergence for all genes with paralogs at the dominant allele *S*-hemizygous regions and their closest paralogs in *Linum grandiflorum* and *Linum perenne*.

S-locus gene	Paralog	Contig	Start	End	d_S[1]	SE[2]
Lgra10G00000021985	Lgra10G00000008224	h1tg000005l	31463522	31465015	0.0168	0.0074
Lgra10G00000021986	Lgra10G00000031222	h1tg000019l	6180865	6181227	0.119	0.033
Lgra10G00000021988	Lgra10G00000028065	h1tg000010l	2169113	2170729	0.396	0.105
Lgra10G00000021991	Lgra10G00000026991	h1tg000006l	29657130	29658098	0.346	0.097
t1STRG16993	Lgra10G00000018032	h1tg000014l	11676715	11686899	0.641	0.065
Lgra10G00000022001	Lgra10G00000009030	h1tg000005l	72849596	72850303	0.773	0.087
Lgra10G00000022006	Lgra10G00000017843	h1tg000014l	4161762	4167805	0.149	0.064
Lgra10G00000022011	Lgra10G00000019904	h1tg000018l	20747387	20748495	0.0459	0.0228
Lgra10G00000022013	Lgra10G00000024773	h1tg000016l	4164938	4167333	0.455	0.038
Lgra10G00000022015	Lgra10G00000018894	h1tg000021l	17187215	17188497	0.000	0.000
Lgra10G00000022016	Lgra10G00000017915	h1tg000014l	7437038	7440734	0.412	0.069
t4STRG14266	Lgra10G00000036514	h1tg000009l	21362014	21366348	0.0604	0.0440
Lgra10G00000022018	Lgra10G00000019815	h1tg000018l	18975568	18975744	0.388	0.082
Lper10G00000044768	Lper10G00000015926	h1tg000818l	27001	32440	0.0109	0.0042
Lper10G00000044771	Lper10G00000041146	h1tg000102l	1490252	1493370	0.371	0.089
Lper10G00000044772	Lper10G00000028692	h1tg000071l	2098032	2105393	0.389	0.091
Lper10G00000044773	agat-gene-1	h1tg000002l	1586118	1586688	0.0710	0.0337
Lper10G00000044780	Lper10G00000015230	h1tg000052l	5534072	5534446	0.0749	0.0339
Lper10G00000044783	Lper10G00000025512	h1tg000059l	1943219	1945331	0.429	0.035
Lper10G00000044784	Lper10G00000016519	h1tg000021l	35147299	35147439	0.000	0.000
Lper10G00000044786	Lper10G00000040280	h1tg000046l	6310639	6313807	0.0243	0.0220
Lper10G00000044789	Lper10G00000036747	h1tg000065l	9284490	9284729	0.707	0.119
Lper10G00000044791	Lper10G00000034992	h1tg000003l	1586489	1590067	0.0690	0.0465
Lper10G00000044793	Lper10G00000044869	h1tg000002l	7081115	7082187	0.0730	0.0307
Lper10G00000044799	Lper10G00000004039	h1tg000051l	13488572	13489794	0.408	0.083
Lper10G00000044801	Lper10G00000041292	h1tg000004l	448836	449093	0.588	0.066
Lper10G00000044805	Lper10G00000003205	h1tg000051l	3100528	3101385	0.619	0.039
Lper10G00000044806	Lper10G00000008228	h1tg000025l	2083158	2083955	0.805	0.047

[1]Synonymous divergence

[2]Standard error

Table S5 Brassinosteroid treatment has no detectable morph-specific effect on stamen length (mm) in *Linum perenne* or *Linum tenue*.

Species	Source of variation[1]	Df[2]	SS[3]	MS[4]	F[5]	*P*-value
L. perenne	Morph	1	31.23	32.23	54.09	<0.0001
	Treatment	1	0.59	0.59	1.02	0.32 (NS)
	Morph*Treatment	1	0.32	0.32	0.55	0.46 (NS)
	Residuals	40	23.09	0.58		
L. tenue	Morph	1	113.3	113.3	417.4	<0.0001
	Treatment	1	1.22	1.22	4.50	0.04
	Morph*Treatment	1	0.009	0.009	0.0334	0.86 (NS)
	Residuals	72	19.55	0.27		

[1]Analysis of variance sources of variation
[2]Degrees of freedom
[3]Sums of squares
[4]Mean squares
[5]F-statistic

Table S6 Analysis of log-transformed mean cell length data from the epibrassinolide supplementation experiment shows that brassinosteroid treatment has a morph-specific effect on style cell length (mm) in *Linum perenne*.

Source of variation[1]	Df[2]	SS[3]	MS[4]	F[5]	*P*-value
Morph	1	3.47	3.47	121.8	<0.0001
Segment	2	1.44	0.72	25.3	<0.0001
Treatment	1	0.66	0.66	23.3	<0.0001
Morph*Segment	2	0.21	0.11	3.7	0.03
Morph*Treatment	1	0.16	0.16	5.7	0.02
Residuals	75	2.13	0.03		

[1]Analysis of variance sources of variation
[2]Degrees of freedom
[3]Sums of squares
[4]Mean squares
[5]F-statistic

Supplementary Notes

Note S1 Assembly contamination screening and masking results.
In *L. perenne* two contigs (h1tg000166c and h1tg000316l) were removed due to high coverage (>200x) and similarity to plastid or mitochondrial DNA, while four regions of 88.4 kb, 59 kb, 186.4 kb, and 78.6 kb were masked in contigs h1tg000014l, h1tg000021l, h1tg000036l, and h1tg000092l, respectively, due to signs of contamination. In *L. grandiflorum*, two contigs (h1tg000046c and h1tg000187c) were removed due to high similarity to plastid or mitochondrial DNA.

Note S2 Population-based validation of GWAS results.
Because family-based GWAS analysis has limitations in terms of resolution, we conducted a follow-up validation analysis based on 53 samples from a natural population. Analyzing a total of 10,681 SNPs in 53 individuals from one natural population of *L. perenne* (population ger3), we identified a total of 220 SNPs on six contigs that were significantly associated with floral morph (FDR<0.05). Only five SNPs mapped to contigs not identified in the family-based analysis, and the remaining 215 SNPs (97.7%) mapped to the same contigs as in the family-based analysis). The two major contigs identified in the family-based analysis harbored 85.5% of the SNPs significantly associated with floral morph, and the length of the associated genomic regions amounted to 32.1 Mb. These results support those of our family-based analysis.

Note S3 Extensive linkage disequilibrium at the *L. perenne* distyly S-locus.
To test for limited recombination in a large genomic region around the *S*-hemizygous region in *L. perenne*, we estimated linkage disequilibrium (LD) across the ~26 Mb *S*-locus contig (h1tg000002l). For this purpose, we analyzed population genomic data from three natural populations of *L. perenne* from Germany (ger2, n=63; ger3, n=53; ger5, n=20; Table S1). We assessed LD by estimating r^2 between each pair of SNPs along the *S*-locus contig as well as for a control contig of similar size (h1tg000031l) in PLINK v1.90b4.9 (Purcell *et al.*, 2007). We aggregated r^2 values in bins of 100 kb and plotted LD heatmaps with these bins for both contigs using *Ldheatmap* v1.0-5 (Shin *et al.*, 2006) in R v.4.4.0. We compared r^2 values for the two contigs using a Wilcoxon rank sum test in R v.4.4.0.

We found elevated LD along the length of the *S*-locus contig, whereas that was not the case for the control contig (Fig. S2), suggesting that the extended LD is not a result of non-equilibrium demographic processes. The extended LD at the *S*-locus in natural populations of *L. perenne* suggests that the genomic region with limited recombination is more extensive in *L. perenne* than in *L. grandiflorum*, where GWAS hits were in the immediate vicinity of the S-hemizygous region (Fig. 2a-b). Likewise, analyses of LD around the *L. tenue S*-locus found no evidence of extended LD outside of the S-hemizygous region (Gutiérrez-Valencia *et al.*, 2022).

Note S4 Description of gene content at the S-loci of *L. grandiflorum and L. perenne*.
The number of annotated genes in the *S*-linked hemizygous region differed greatly between *L. tenue*, *L. grandiflorum* and *L. perenne,* with the longer, dominant haplotype having only nine protein-coding genes in *L. tenue* (Gutiérrez-Valencia *et al.*, 2022), in contrast to those of *L.*

grandiflorum and *L. perenne,* which harbored 24 and 32 protein-coding genes, respectively. In *L. grandiflorum*, the thrum-hemizygous region harbored nine genes with functional annotation, including *TSS1*, *WDR-44*, *MPT1*, one gene involved in auxin-mediated developmental responses (*NOV1*) and five additional genes with various functions not clearly related to floral development or reproduction (Fig. 4a; Data S1B). Two of the genes present on the dominant *S*-haplotype (*NOV* and *4CLL6*) were also present on the recessive *S*-haplotype. The remaining 15 protein-coding genes were of unknown function and five of them overlapped to more than 50% with annotated repeats (Fig. 4a; Data S1B).

In *L. perenne* the longer, dominant *S*-haplotype harbored 12 genes with functional annotation, including *TSS1*, *WDR-44*, *MPT1*, as well as two genes with functions related to floral or reproductive development: two MADS-box transcription factors (*AGL8* and *AGL80*) involved in floral meristem development and female gametophyte cell differentiation, respectively (Fig. 4b; Data S1B). The remaining functionally annotated genes had no clear connection to floral morphology or reproduction, and 14 out of 20 genes without functional annotation showed more than 50% overlap with annotated repeats (Data S1B).

Note S5 Comparative molecular evolutionary analysis of rates of evolution of TSS1 and WDR-44.

We tested whether rates of evolution (d_N/d_S) at *TSS1* and *WDR-44* were elevated in relation to those of other genes across the genomes of *L. grandiflorum, L. perenne* and *L. tenue*. To do so, we compared estimates of d_N/d_S at *TSS1* and *WDR-44* in *L. grandiflorum, L. perenne* and *L. tenue* to those at a set of 3,864 1-1-1 orthologs, identified using OrthoFinder v2.5.5 (Emms and Kelly, 2019). We aligned the orthologs with PRANK (Löytynoja 2013) and we found no evidence for more extreme d_N/d_S values at either *TSS1* or *WDR-44* compared to 1-1-1 orthologs, consistent with purifying selection (mean d_N/d_S for 1-1-1 orthologs: 0.18, 95% CI 0.05-0.41, for *TSS1*: 0.32, for *WDR-44*: 0.35).

References

Emms DM, Kelly S. 2019. OrthoFinder: Phylogenetic orthology inference for comparative genomics. *Genome Biology* 20: 238.

Gutiérrez-Valencia J, Fracassetti M, Berdan EL, Bunikis I, Soler L, Dainat J, Kutschera VE, Losvik A, Désamoré A, Hughes PW, *et al.* 2022. Genomic analyses of the *Linum* distyly supergene reveal convergent evolution at the molecular level. *Current Biology* 32: 4360-4371.e6

Löytynoja A. 2013. Phylogeny-aware alignment with PRANK. In *Multiple sequence alignment methods* (pp. 155-170). Totowa, NJ: Humana Press.

Purcell S, Neale B, Todd-Brown K, Thomas L, Ferreira MAR, Bender D, Maller J, Sklar P, De Bakker PIW, Daly MJ, *et al.* 2007. PLINK: A tool set for whole-genome association and population-based linkage analyses. *American Journal of Human Genetics* 81: 559–575.

Shin J-H, Blay S, McNeney B, Graham J. 2006. LDheatmap: An R Function for Graphical Display of Pairwise Linkage Disequilibria Between Single Nucleotide Polymorphisms. *JSS Journal of Statistical Software* 16: 1–9.
